# The Rising Tide of Coronary Crisis: Decoding Age‐Specific Disparities in Ischemic Heart Disease Burden Through the Global Burden of Disease Study 2021 Revelations: An Ecological Study

**DOI:** 10.1002/hsr2.71244

**Published:** 2025-10-15

**Authors:** Yemin Wang, Jing Li, Hazrat Bilal, Xiao Yu, Lei Sun

**Affiliations:** ^1^ Department of Pathology and Forensic Medicine, College of Basic Medical Sciences Dalian Medical University Dalian Liaoning Province China

**Keywords:** age‐specific trends, future burden projections, gender differences, ischemic heart disease, socio‐demographic index, targeted risk strategies

## Abstract

**Background and Aim:**

Ischemic heart disease (IHD) remains a critical global public health challenge, yet comparative studies on its burden between younger (20–54 years) and older (55+ years) adults are limited. This study aims to quantify differential IHD burden trajectories across these age groups globally, thereby informing tailored prevention strategies.

**Methods:**

Using data from the Global Burden of Disease Study 2021 (GBD), we evaluated the IHD burden through prevalence, disability‐adjusted life years (DALYs), and mortality. Trends from 1990 to 2021 were analyzed via estimated annual percentage change (EAPC), and proportional risk contributions were assessed via population attribution scores. Future trends were projected for the next 30 years via a Bayesian age‐period‐cohort model.

**Results:**

From 1990 to 2021, the global DALY rates and mortality rates decreased, whereas the prevalence increased, particularly among younger adults (EAPC = 0.98 [0.93–1.02]). Males presented a greater IHD burden than females did. Low‐ and medium‐socio‐demographic index (SDI) regions experienced greater disease burdens than high‐SDI regions did (EAPC = 1.63 [1.57–1.68]). Metabolic and behavioral risk factors, including high low‐density lipoprotein (LDL) cholesterol (52.74%) and tobacco use (33.90%), are predominant among younger adults, whereas older adults face greater risks from high blood pressure (53.74%) and elevated fasting glucose (16.88%). Projections suggest that mortality will decline in both age groups (20–54 years: 22.92/100,000 population [95% UI: 17.73–28.11]; ≥ 55 years: 404.28/100,000 population [95% UI: 274.18–534.35]), whereas the prevalence will stabilize in older adults but rise and later decline in younger populations.

**Conclusion:**

The global IHD burden was associated with decreasing mortality and DALY rates but increasing prevalence, with notable disparities by gender, SDI, and region. Prioritizing age‐specific and targeted interventions is crucial to reversing the prevalence trend and sustaining mortality reductions, ultimately mitigating the global IHD burden.

## 1 Introduction

Ischemic heart disease (IHD), a leading cause of global morbidity and mortality [1], arises from myocardial hypoxia due to insufficient coronary blood flow [2, 3]. Despite advancements in cardiovascular care, IHD remains a critical public health threat, with 9.14 million deaths and 197 million cases reported in 2019 [4]. Concerningly, its burden is rising alongside demographic shifts and lifestyle changes, even as COVID‐19 dominates global health agendas [5].

Although IHD predominantly affects older adults, increasing evidence suggests a trend toward a younger age of onset, with early‐onset IHD incidence increasing in various regions worldwide [6, 7]. The distinct lifestyles and social environments of younger and older individuals likely contribute to differences in their clinical features and prognoses. While previous studies have examined the IHD burden at the national or regional level [1, 3, 8], there remains a significant lack of comparative analyses focusing separately on the epidemiological characteristics and trends in younger and older populations. In particular, critical gaps persist regarding the dynamic changes in disease burden across different age groups, global disparities revealed through stratified analyses, heterogeneity in risk factor‐driven disease patterns, and multidimensional trend predictions. Furthermore, while traditional cardiovascular risk factors such as diabetes and hypertension are well‐established contributors to IHD burden [9, 10], young adults exhibit unique risk profiles compared with their older counterparts. Given these disparities, a systematic and in‐depth analysis of the IHD burden across age groups is imperative. This includes comparative epidemiological assessments and evaluations of age‐specific risk factors to inform targeted and precise prevention strategies. Moreover, given the dynamic shifts in disease patterns, it is essential not only to gain a comprehensive understanding of the current state but also to incorporate forward‐looking projections. In particular, long‐term forecasts over the next 30 years based on different age groups are vital for guiding the allocation of IHD resources and setting healthcare priorities.

Our study aims to analyze global, regional, and national variations in IHD burden from 1990 to 2021 using age‐stratified (20–54 vs. 55+ years) prevalence, mortality, and disability‐adjusted life years (DALYs) metrics via the Global Burden of Disease Study (GBD) database. It evaluates socioeconomic gradients in disease patterns across both age groups, quantifies age‐specific contributions of behavioral and metabolic risk factors to mortality/DALYs, and applies advanced modeling to forecast 30‐year burden trajectories. Findings aim to inform precision public health strategies targeting IHD prevention across life course stages and resource settings.

## 2 Methods

### 2.1 Study Population and Data Collection

This study utilized the GBD 2021 database to analyze epidemiological trends in IHD. Age‐specific data on prevalence, mortality, and DALYs were extracted via the Global Health Data Exchange (GHDx) platform. The study population was divided into two age groups: younger adults (20–54 years) and older adults (55+ years), aligning with demographic and clinical relevance in IHD research.

### 2.2 Socio‐Demographic Index (SDI)

Countries and regions were stratified into five SDI categories (low, low‐middle, middle, high‐middle, high) based on fertility, education, and income metrics [5]. The SDI framework enabled exploration of socioeconomic disparities in IHD burdens between younger and older populations.

### 2.3 Bayesian Age‐Period‐Cohort (BAPC) Analysis

To project the future burden of IHD, we employed the BAPC model. This model operates under the assumption that the effects of age, period, and cohort on disease incidence are independent yet interact multiplicatively, creating a synergistic effect on disease trends [11]. This assumption allows the model to flexibly capture variations in disease patterns across different age groups, time periods, and cohorts. Projections for the next 30 years were generated using R (version 4.4.1) software.

### 2.4 Statistical Analysis

Trends in prevalence, mortality, and DALYs (1990–2021) were assessed via estimated annual percentage change (EAPC). EAPC was derived from linear regression of the natural logarithm of rates over time [12], with significance set at a two‐sided *p*‐value < 0.05. 95% Uncertainty intervals (UIs) were calculated using the 2.5th and 97.5th percentiles of 1000 GBD‐generated estimates. All analyses were performed in R (version 4.4.1).

### 2.5 Ethical Approval and Consent to Participate

This study received approval from Dalian Medical University's Ethics Committee (Approval No. DMU‐IRB‐2025‐027). Informed consent was waived as all data were anonymized and non‐identifiable.

## 3 Results

### 3.1 Global Comparative Burden Analysis

Global estimates revealed a pronounced age disparity in IHD burden in 2021, with significantly higher case numbers, DALYs, and deaths in adults aged ≥ 55 years compared to those aged 20–54 years (Tables 1 and 2).

**Table 1:** Ischemic heart disease burden in people aged 20–54 years across regions during 1990–2021.

	Prevalence					DALYs					Deaths				
	1990		2021		1990–2021	1990		2021		1990–2021	1990		2021		1990–2021
Location	Number	Rate (per 100,000)	Number	Rate (per 100,000)	EAPC	Number	Rate (per 100,000)	Number	Rate (per 100,000)	EAPC	Number	Rate (per 100,000)	Number	Rate (per 100,000)	EAPC
Global	19,807,122.39 (16,915,553.47–23,322,371.69)	824.05 (703.75–970.30)	40,741,428.40 (33,729,294.99–50,035,950.55)	1080.81 (894.79–1327.38)	0.98 (0.93–1.02)	30,641,784.56 (29,313,357.73–31,956,855.97)	1274.82 (1219.55–1329.53)	44,507,120.07 (41,975,648.24–47,219,100.13)	1180.71 (1113.55–1252.65)	−0.31 (−0.40 to −0.22)	670,338.57 (642,923.82–698,512.04)	27.89 (26.75–29.06)	979,689.56 (921,164.64–1,041,696.54)	25.99 (24.44–27.63)	−0.27 (−0.36 to −0.18)
SDI quintile															
High SDI	3,239,968.38 (2,809,555.31–3,725,845.29)	733.44 (636.01–843.43)	3,997,057.60 (3,381,229.02–4,723,451.88)	774.16 (654.88–914.84)	−0.04 (−0.12–0.05)	4,557,091.37 (4,475,293.55–4,640,349.43)	1031.60 (1013.08–1050.45)	3,203,390.22 (3,009,256.35–3,425,415.08)	620.44 (582.84–663.44)	−1.71 (−1.80 to −1.63)	103,055.72 (101,351.76–104,805.33)	23.33 (22.94–23.73)	72,056.79 (68,038.87–76,674.68)	13.96 (13.18–14.85)	−1.72 (−1.82 to −1.62)
High‐middle SDI	5,005,773.88 (4,283,679.40–5,908,254.18)	961.09 (822.45–1134.37)	8,568,950.83 (7,038,510.86–10,522,260.54)	1310.25 (1076.24–1608.93)	1.12 (1.00–1.24)	7,193,217.18 (6,838,649.62–7,509,843.88)	1381.08 (1313.00–1441.87)	6,711,381.66 (6,148,012.75–7,388,722.64)	1026.22 (940.08–1129.79)	−1.41 (−1.80 to −1.01)	160,073.47 (152,598.33–167,032.68)	30.73 (29.30–32.07)	150,207.14 (136,867.61–165,391.39)	22.97 (20.93–25.29)	−1.35 (−1.76 to −0.94)
Middle SDI	5,829,113.26 (4,936,211.72–6,965,577.83)	742.76 (628.98–887.57)	14,313,450.40 (11,703,206.85–17,787,040.34)	1164.17 (951.87–1446.70)	1.63 (1.57–1.68)	9,057,274.40 (8,556,566.34–9,539,173.59)	1154.10 (1090.30–1215.50)	15,567,797.98 (14,531,838.76–16,680,959.59)	1266.20 (1181.94–1356.73)	0.39 (0.34–0.43)	194,028.39 (183,629.99–204,375.57)	24.72 (23.40–26.04)	342,530.46 (319,734.07–367,352.67)	27.86 (26.01–29.88)	0.50 (0.45–0.54)
Low‐middle SDI	4,332,687.94 (3,682,833.22–5,101,438.88)	922.66 (784.27–1086.37)	10,311,731.61 (8,505,859.64–12,536,715.29)	1126.18 (928.95–1369.17)	0.80 (0.73–0.87)	7,763,889.31 (7,140,440.36–8,447,003.63)	1653.35 (1520.58–1798.82)	14,721,652.76 (13,536,284.60–15,977,207.23)	1607.80 (1478.34–1744.92)	0.08 (−0.00–0.15)	167,560.55 (154,150.89–182,117.86)	35.68 (32.83–38.78)	321,556.03 (295,281.94–349,402.64)	35.12 (32.25–38.16)	0.14 (0.05–0.23)
Low SDI	1,377,510.96 (1,180,899.46–1,605,389.64)	746.96 (640.34–870.52)	3,517,162.56 (2,922,032.68–4,208,968.41)	779.65 (647.73–933.01)	0.17 (0.07–0.26)	2,029,516.37 (1,780,524.04–2,348,647.13)	1100.51 (965.49–1273.56)	4,265,099.63 (3,796,957.27–4,771,318.95)	945.45 (841.68–1057.66)	−0.61 (−0.70 to −0.53)	44,712.20 (39,379.83–51,848.64)	24.25 (21.35–28.12)	92,499.05 (82,404.57–103,604.90)	20.50 (18.27–22.97)	−0.67 (−0.77 to −0.57)
Region															
East Asia	4,216,289.68 (3,494,283.58–5,121,880.23)	693.07 (574.39–841.93)	9,347,080.35 (7,481,577.59–11,849,841.72)	1270.50 (1016.93–1610.68)	2.14 (1.98–2.29)	4,423,183.90 (3,862,165.39–5,029,240.82)	727.08 (634.86–826.71)	6,256,673.99 (5,110,018.14–7,535,650.90)	850.44 (694.58–1024.28)	0.70 (0.55–0.85)	93,563.03 (81,381.34–106,563.41)	15.38 (13.38–17.52)	138,266.47 (111,760.59–168,068.70)	18.79 (15.19–22.84)	0.88 (0.71–1.04)
Southeast Asia	911,618.65 (782,169.66–1,058,753.88)	448.49 (384.80–520.88)	2,230,321.97 (1,862,021.35–2,660,640.94)	629.18 (525.28–750.58)	1.34 (1.25–1.42)	2,665,411.67 (2,428,680.92–2,905,234.32)	1311.30 (1194.84–1429.29)	5,196,197.04 (4,570,281.68–5,946,124.58)	1465.87 (1289.30–1677.43)	0.51 (0.45–0.56)	56,506.48 (51,438.49–61,575.71)	27.80 (25.31–30.29)	113,814.68 (99,749.85–130,218.21)	32.11 (28.14–36.74)	0.65 (0.59–0.71)
Oceania	16,058.71 (14,394.63–18,058.16)	594.22 (532.65–668.21)	43,860.03 (37,762.52–50,478.95)	695.41 (598.74–800.36)	0.64 (0.59–0.68)	61,340.30 (48,307.78–78,855.66)	2269.79 (1787.54–2917.91)	145,122.99 (115,911.80–179,916.10)	2300.97 (1837.82–2852.63)	0.13 (0.06–0.20)	1351.01 (1068.02–1742.27)	49.99 (39.52–64.47)	3197.00 (2557.00–3964.44)	50.69 (40.54–62.86)	0.15 (0.08–0.21)
Central Asia	288,994.84 (258,533.61–323,123.48)	971.94 (869.49–1086.72)	545,980.56 (475,634.71–622,283.24)	1170.77 (1019.92–1334.39)	1.03 (0.83–1.23)	678,395.88 (644,622.19–710,550.98)	2281.56 (2167.97–2389.70)	703,040.42 (617,928.96–792,192.02)	1507.56 (1325.05–1698.73)	−1.92 (−2.37 to −1.46)	15,134.59 (14,435.97–15,839.79)	50.90 (48.55–53.27)	15,630.39 (13,731.38–17,673.80)	33.52 (29.44–37.90)	−1.81 (−2.25 to −1.36)
Central Europe	741,584.04 (647,720.14–842,883.11)	1250.49 (1092.22–1421.31)	708,728.69 (601,986.64–823,998.05)	1296.17 (1100.95–1506.98)	−0.19 (−0.31 to −0.08)	1,373,975.01 (1,347,676.47–1,396,382.21)	2316.86 (2272.52–2354.65)	530,895.34 (487,100.22–575,412.51)	970.94 (890.84–1052.35)	−3.32 (−3.50 to −3.14)	31,020.70 (30,432.79–31,510.59)	52.31 (51.32–53.13)	12,177.84 (11,150.22–13,216.74)	22.27 (20.39–24.17)	−3.27 (−3.46 to −3.07)
Eastern Europe	1,775,106.32 (1,467,012.27–2,158,548.70)	1609.01 (1329.75–1956.58)	1,907,648.31 (1,521,288.38–2,409,192.99)	1936.29 (1544.13–2445.37)	0.70 (0.49–0.90)	2,897,504.12 (2,792,152.65–2,981,910.31)	2626.39 (2530.89–2702.90)	1,977,885.18 (1,778,631.06–2,194,571.07)	2007.59 (1805.34–2227.52)	−1.82 (−2.64 to −1.01)	66,596.12 (64,276.49–68,473.66)	60.36 (58.26–62.07)	45,210.10 (40,555.31–50,317.66)	45.89 (41.16–51.07)	−1.75 (−2.58 to −0.92)
High‐income Asia Pacific	310,918.39 (259,008.75–371,947.85)	352.97 (294.04–422.26)	305,811.29 (251,057.97–363,827.10)	363.39 (298.33–432.33)	−0.22 (−0.38 to −0.06)	400,537.44 (382,238.73–420,426.25)	454.72 (433.94–477.30)	261,094.41 (250,765.02–271,944.50)	310.26 (297.98–323.15)	−1.32 (−1.47 to −1.16)	8751.21 (8366.34–9184.89)	9.93 (9.50–10.43)	5836.49 (5628.28–6052.50)	6.94 (6.69–7.19)	−1.24 (−1.40 to −1.09)
Australasia	70,792.31 (62,330.82–79,472.84)	703.45 (619.37–789.71)	98,232.16 (81,637.47–116,198.56)	673.50 (559.72–796.68)	−0.30 (−0.36 to −0.25)	92,749.12 (89,548.92–96,499.82)	921.63 (889.83–958.90)	53,646.32 (49,907.98–57,199.48)	367.81 (342.18–392.17)	−2.97 (−3.07 to −2.86)	2113.21 (2035.85–2192.91)	21.00 (20.23–21.79)	1211.98 (1128.29–1292.48)	8.31 (7.74–8.86)	−2.97 (−3.07 to −2.88)
Western Europe	1,349,162.45 (1,196,952.71–1,527,622.80)	714.01 (633.46–808.46)	1,306,908.56 (1,097,206.09–1,534,900.90)	664.94 (558.25–780.95)	−0.18 (−0.23 to −0.13)	1,840,021.52 (1,810,122.26–1,869,719.51)	973.79 (957.97–989.51)	732,275.68 (709,743.07–754,842.11)	372.58 (361.11–384.06)	−3.01 (−3.08 to −2.94)	41,927.90 (41,300.45–42,510.20)	22.19 (21.86–22.50)	16,649.00 (16,190.56–17,068.23)	8.47 (8.24–8.68)	−3.00 (−3.08 to −2.93)
Southern Latin America	119,054.80 (105,978.29–134,756.90)	535.38 (476.57–605.99)	183,936.53 (157,799.12–212,409.96)	549.58 (471.48–634.65)	−0.02 (−0.05–0.01)	236,319.45 (227,178.71–245,029.04)	1062.70 (1021.60–1101.87)	155,799.98 (149,083.48–162,884.01)	465.51 (445.44–486.68)	−2.52 (−2.67 to −2.37)	5318.44 (5112.22–5519.67)	23.92 (22.99–24.82)	3490.89 (3338.58–3638.61)	10.43 (9.98–10.87)	−2.53 (−2.66 to −2.40)
High‐income North America	1,148,767.46 (938,024.79–1,417,853.20)	810.41 (661.74–1000.24)	996,287.43 (810,945.57–1,223,319.95)	592.74 (482.47–727.81)	−1.39 (−1.63 to −1.15)	1,585,056.89 (1,565,001.63–1,604,410.03)	1118.19 (1104.05–1131.85)	1,231,847.07 (1,185,915.96–1,271,390.18)	732.88 (705.56–756.41)	−1.47 (−1.72 to −1.22)	36,136.01 (35,759.66–36,493.31)	25.49 (25.23–25.74)	28,339.35 (27,320.13–29,220.00)	16.86 (16.25–17.38)	−1.41 (−1.67 to −1.15)
Caribbean	159,038.28 (142,639.02–177,680.92)	1001.26 (898.02–1118.63)	298,554.33 (257,593.21–342,844.63)	1301.59 (1123.01–1494.68)	0.99 (0.95–1.03)	220,149.50 (207,361.54–234,679.07)	1386.00 (1305.49–1477.47)	285,974.03 (240,315.57–341,502.19)	1246.74 (1047.69–1488.83)	−0.10 (−0.33–0.13)	4859.12 (4565.97–5196.94)	30.59 (28.75–32.72)	6372.55 (5344.23–7627.48)	27.78 (23.30–33.25)	−0.06 (−0.29–0.17)
Andean Latin America	92,237.21 (82,486.49–103,369.02)	588.19 (526.01–659.18)	260,408.47 (223,443.06–300,509.65)	799.94 (686.39–923.13)	1.14 (1.08–1.19)	117,927.14 (105,461.13–133,014.68)	752.02 (672.52–848.23)	166,626.48 (137,165.73–200,625.34)	511.86 (421.36–616.30)	−1.39 (−1.76 to −1.03)	2431.39 (2163.70–2751.20)	15.50 (13.80–17.54)	3457.53 (2800.47–4226.09)	10.62 (8.60–12.98)	−1.38 (−1.75 to −1.01)
Central Latin America	500,304.24 (433,677.31–579,219.52)	733.51 (635.83–849.21)	1,228,222.05 (1,028,135.41–1,485,029.27)	982.97 (822.83–1188.49)	1.02 (0.99–1.05)	568,895.91 (558,088.11–578,533.29)	834.08 (818.23–848.21)	1,161,652.40 (1,036,977.70–1,305,433.78)	929.69 (829.91–1044.76)	0.25 (−0.05–0.56)	12,172.43 (11,934.33–12,367.65)	17.85 (17.50–18.13)	25,322.46 (22,512.80–28,510.42)	20.27 (18.02–22.82)	0.29 (−0.00–0.59)
Tropical Latin America	445,276.00 (370,045.28–532,778.22)	653.39 (543.00–781.79)	938,076.42 (773,877.47–1,156,444.53)	804.25 (663.48–991.47)	0.67 (0.64–0.70)	865,496.27 (846,059.90–883,223.15)	1270.01 (1241.49–1296.03)	1,018,579.65 (981,280.98–1,054,190.71)	873.27 (841.29–903.80)	−1.25 (−1.33 to −1.18)	19,077.99 (18,669.83–19,451.24)	27.99 (27.40–28.54)	22,566.23 (21,726.17–23,319.23)	19.35 (18.63–19.99)	−1.23 (−1.29 to −1.16)
North Africa and Middle East	1,972,966.16 (1,772,063.16–2,198,302.34)	1470.65 (1320.90–1638.62)	5,962,991.61 (5,162,926.79–6,824,356.69)	1921.84 (1663.99–2199.46)	0.86 (0.75–0.97)	3,192,451.09 (2,950,503.11–3,505,064.06)	2379.66 (2199.31–2612.68)	5,501,030.26 (4,696,283.36–6,395,895.31)	1772.96 (1513.59–2061.37)	−1.02 (−1.09 to −0.95)	69,236.38 (63,964.47–76,075.22)	51.61 (47.68–56.71)	120,340.72 (102,557.44–140,440.21)	38.79 (33.05–45.26)	−0.99 (−1.07 to −0.92)
South Asia	4,678,081.21 (3,832,219.66–5,680,406.23)	1025.80 (840.32–1245.59)	11,605,414.23 (9,234,775.89–14,655,693.30)	1268.65 (1009.51–1602.10)	0.84 (0.76–0.91)	8,276,222.02 (7,518,362.51–9,108,007.94)	1814.79 (1648.61–1997.18)	16,486,121.42 (14,981,280.67–17,900,750.16)	1802.19 (1637.69–1956.83)	0.11 (0.01–0.21)	179,094.34 (163,212.20–197,269.07)	39.27 (35.79–43.26)	360,659.59 (326,761.16–393,075.45)	39.43 (35.72–42.97)	0.17 (0.06–0.27)
Central Sub‐Saharan Africa	104,830.84 (93,171.73–116,932.16)	518.60 (460.92–578.47)	295,515.07 (253,882.02–339,950.21)	543.79 (467.18–625.55)	0.24 (0.19–0.29)	158,631.77 (118,305.05–210,989.39)	784.75 (585.26–1043.77)	385,895.52 (286,745.36–509,593.83)	710.10 (527.65–937.72)	−0.33 (−0.41 to −0.25)	3592.33 (2662.86–4763.87)	17.77 (13.17–23.57)	8645.73 (6351.47–11,439.81)	15.91 (11.69–21.05)	−0.34 (−0.43 to −0.26)
Eastern Sub‐Saharan Africa	344,786.69 (297,348.54–398,794.26)	508.82 (438.81–588.52)	946,288.76 (797,797.54–1,112,086.35)	551.85 (465.25–648.54)	0.24 (0.17–0.31)	391,531.14 (342,947.04–472,743.35)	577.80 (506.11–697.65)	934,945.26 (792,620.61–1,099,523.06)	545.24 (462.24–641.21)	−0.44 (−0.54 to −0.35)	8361.45 (7297.73–10,118.41)	12.34 (10.77–14.93)	19,635.53 (16,621.95–23,169.49)	11.45 (9.69–13.51)	−0.50 (−0.60 to −0.41)
Southern Sub‐Saharan Africa	147,498.94 (123,092.23–176,295.67)	685.00 (571.65–818.74)	314,874.81 (255,798.04–390,016.87)	801.13 (650.82–992.31)	0.37 (0.30–0.45)	173,834.19 (159,762.98–188,110.32)	807.30 (741.96–873.60)	282,524.58 (253,557.81–316,735.08)	718.82 (645.12–805.86)	−0.37 (−0.83–0.10)	3711.24 (3399.99–4019.30)	17.24 (15.79–18.67)	6234.14 (5556.57–6992.80)	15.86 (14.14–17.79)	−0.23 (−0.68–0.22)
Western Sub‐Saharan Africa	413,755.18 (355,277.07–480,426.26)	580.88 (498.78–674.48)	1,216,286.77 (1,016,337.00–1,456,551.89)	643.17 (537.44–770.22)	0.42 (0.36–0.49)	422,150.25 (338,459.14–505,398.96)	592.67 (475.17–709.54)	1,039,292.03 (822,268.38–1,249,460.38)	549.57 (434.81–660.71)	−0.24 (−0.38 to −0.09)	9383.20 (7489.07–11,230.18)	13.17 (10.51–15.77)	22,630.90 (17,915.21–27,396.44)	11.97 (9.47–14.49)	−0.31 (−0.45 to −0.17)

Abbreviations: DALYs, disability‐adjusted life years; EAPC, estimated annual percentage change; SDI, socio‐demographic index.

**Table 2:** Ischemic heart disease burden in people aged 55+ years across regions during 1990–2021.

	Prevalence					DALYs					Deaths				
	1990		2021		1990–2021	1990		2021		1990–2021	1990		2021		1990–2021
Location	Number	Rate (per 100,000)	Number	Rate (per 100,000)	EAPC	Number	Rate (per 100,000)	Number	Rate (per 100,000)	EAPC	Number	Rate (per 100,000)	Number	Rate (per 100,000)	EAPC
Global	92,289,382.35 (81,067,796.91–105,262,046.95)	13,745.29 (12,073.98–15,677.40)	213,453,241.21 (184,076,211.92–249,859,637.02)	14,364.42 (12,387.48–16,814.40)	0.05 (0.01–0.09)	87,824,595.65 (83,702,049.38–90,883,694.86)	13,080.32 (12,466.32–13,535.93)	143,185,414.92 (133,394,005.19–151,040,815.63)	9635.72 (8976.80–10,164.35)	−1.12 (−1.19 to −1.05)	4,687,253.21 (4,414,722.00–4,860,810.70)	698.10 (657.51–723.95)	8,002,785.77 (7,312,844.59–8,503,729.13)	538.55 (492.12–572.26)	−0.93 (−1.00 to −0.86)
SDI quintile															
High SDI	21,999,850.71 (19,266,297.61–24,944,436.88)	11,798.49 (10,332.48–13,377.66)	30,227,014.34 (26,295,362.06–35,004,963.84)	8761.12 (7621.56–10,145.98)	−1.26 (−1.43 to −1.09)	27,421,347.69 (25,737,624.37–28,260,872.52)	14,706.03 (13,803.05–15,156.26)	20,331,818.06 (18,353,633.84–21,447,304.84)	5893.06 (5319.69–6216.37)	−3.25 (−3.40 to −3.10)	1,628,965.11 (1,488,037.09–1,693,327.45)	873.61 (798.03–908.13)	1,320,158.44 (1,143,293.57–1,414,389.00)	382.64 (331.38–409.95)	−2.98 (−3.11 to −2.84)
High‐middle SDI	25,723,348.04 (22,674,794.40–29,178,497.76)	14,909.95 (13,142.93–16,912.65)	55,244,951.26 (47,424,484.20–64,705,531.19)	15,935.56 (13,679.72–18,664.49)	0.08 (−0.03–0.19)	26,266,119.99 (25,195,002.75–27,059,184.97)	15,224.55 (14,603.71–15,684.24)	37,786,326.10 (34,730,709.70–40,727,348.45)	10,899.57 (10,018.17–11,747.91)	−1.37 (−1.67 to −1.07)	1,443,286.67 (1,369,916.90–1,488,494.30)	836.57 (794.04–862.77)	2,299,607.02 (2,075,111.74–2,486,156.97)	663.33 (598.57–717.14)	−0.94 (−1.21 to −0.67)
Middle SDI	22,055,909.64 (19,231,339.58–25,436,778.44)	12,707.91 (11,080.48–14,655.85)	71,030,881.59 (61,016,917.99–84,222,279.94)	15,117.50 (12,986.23–17,925.03)	0.54 (0.48–0.60)	17,203,258.04 (16,202,654.76–18,271,163.97)	9911.96 (9335.45–10,527.26)	45,468,501.94 (41,862,467.25–48,770,794.10)	9677.06 (8909.59–10,379.89)	−0.07 (−0.13 to −0.01)	841,287.33 (787,398.58–894,057.22)	484.72 (453.67–515.13)	2,465,761.36 (2,232,696.45–2,654,271.84)	524.79 (475.18–564.91)	0.33 (0.25–0.41)
Low‐middle SDI	17,150,840.16 (14,991,660.83–19,685,643.25)	17,014.58 (14,872.55–19,529.24)	44,862,747.39 (38,414,745.00–52,713,662.80)	18,608.84 (15,934.24–21,865.36)	0.32 (0.30–0.34)	12,681,419.92 (11,776,525.81–13,528,382.21)	12,580.67 (11,682.96–13,420.90)	31,064,868.35 (28,843,166.17–33,159,099.09)	12,885.55 (11,964.00–13,754.22)	0.17 (0.12–0.22)	582,395.83 (537,077.64–623,970.67)	577.77 (532.81–619.01)	1,509,310.07 (1,398,963.35–1,611,109.52)	626.05 (580.28–668.28)	0.38 (0.33–0.44)
Low SDI	5,238,138.41 (4,573,141.46–5,974,816.97)	14,040.25 (12,257.80–16,014.83)	11,891,101.57 (10,095,825.05–14,014,580.44)	14,491.10 (12,303.28–17,078.87)	0.06 (0.03–0.09)	4,104,317.85 (3,632,667.30–4,558,203.43)	11,001.17 (9736.96–12,217.76)	8,384,377.81 (7,617,503.70–9,322,450.67)	10,217.63 (9283.07–11,360.81)	−0.23 (−0.31 to −0.16)	183,260.09 (162,510.69–202,981.23)	491.21 (435.59–544.07)	399,206.87 (362,780.31–439,680.14)	486.49 (442.10–535.82)	0.05 (−0.04–0.13)
Region															
East Asia	16,070,428.88 (13,733,423.21–18,778,718.39)	10,788.92 (9219.97–12,607.14)	56,019,186.97 (46,742,202.89–67,844,269.40)	14,286.21 (11,920.36–17,301.88)	0.87 (0.76–0.98)	9,576,774.18 (8,486,007.51–10,764,404.25)	6429.39 (5697.10–7226.71)	30,466,059.73 (25,510,618.95–35,389,053.08)	7769.56 (6505.81–9025.04)	0.94 (0.69–1.20)	474,600.39 (421,538.13–532,773.51)	318.62 (283.00–357.68)	1,868,959.12 (1,566,239.82–2,173,053.63)	476.63 (399.43–554.18)	1.68 (1.40–1.97)
Southeast Asia	3,742,100.75 (3,306,299.05–4,245,825.47)	8837.93 (7808.67–10,027.60)	10,697,296.84 (9,330,979.51–12,384,159.26)	9338.16 (8145.44–10,810.70)	0.16 (0.06–0.26)	4,092,722.21 (3,689,866.87–4,490,439.82)	9666.01 (8714.56–10,605.32)	10,646,731.03 (9,570,229.03–11,585,163.50)	9294.02 (8354.29–10,113.22)	−0.16 (−0.28 to −0.04)	195,054.55 (175,393.28–214,233.58)	460.67 (414.24–505.97)	523,676.89 (473,047.93–567,186.09)	457.14 (412.95–495.12)	−0.05 (−0.21–0.11)
Oceania	55,439.60 (49,918.28–61,405.55)	11,523.79 (10,376.12–12,763.89)	149,157.46 (132,933.07–168,372.84)	12,085.70 (10,771.10–13,642.66)	0.10 (0.03–0.18)	74,689.10 (62,684.36–89,960.12)	15,525.03 (13,029.70–18,699.30)	180,629.37 (151,949.14–212,457.50)	14,635.76 (12,311.90–17,214.68)	−0.18 (−0.25 to −0.10)	3170.33 (2681.83–3785.67)	658.99 (557.45–786.90)	7922.59 (6747.40–9314.34)	641.94 (546.72–754.71)	−0.10 (−0.21–0.02)
Central Asia	1,535,792.56 (1,420,391.64–1,671,452.47)	19,202.09 (17,759.23–20,898.26)	2,813,253.95 (2,546,858.82–3,102,216.19)	19,335.36 (17,504.44–21,321.39)	0.05 (−0.17–0.26)	2,122,093.94 (2,027,593.86–2,188,327.89)	26,532.65 (25,351.11–27,360.77)	2,966,175.40 (2,699,031.72–3,260,216.58)	20,386.39 (18,550.32–22,407.32)	−1.27 (−1.76 to −0.77)	116,524.63 (109,385.66–120,752.08)	1456.91 (1367.65–1509.77)	159,690.30 (144,655.91–174,996.80)	1097.54 (994.21–1202.74)	−1.14 (−1.61 to −0.66)
Central Europe	4,767,270.60 (4,223,058.98–5,365,574.95)	17,975.89 (15,923.84–20,231.91)	6,378,996.29 (5,569,995.12–7,276,556.19)	17,227.49 (15,042.65–19,651.49)	−0.48 (−0.61 to −0.35)	6,063,698.79 (5,880,480.25–6,186,157.83)	22,864.31 (22,173.45–23,326.07)	4,992,280.16 (4,582,748.47–5,316,613.92)	13,482.44 (12,376.44–14,358.36)	−2.10 (−2.23 to −1.96)	333,001.13 (318,792.19–340,466.38)	1255.64 (1202.07–1283.79)	319,089.04 (288,518.75–339,728.23)	861.75 (779.19–917.49)	−1.54 (−1.66 to −1.42)
Eastern Europe	10,454,779.18 (9,107,584.53–12,000,126.18)	21,383.05 (18,627.65–24,543.73)	15,507,312.89 (13,126,393.31–18,402,007.44)	24,979.96 (21,144.66–29,642.89)	0.40 (0.21–0.60)	12,786,862.33 (12,335,434.83–13,033,091.57)	26,152.84 (25,229.54–26,656.45)	14,367,404.34 (12,989,595.67–15,690,412.36)	23,143.74 (20,924.30–25,274.91)	−0.84 (−1.39 to −0.28)	719,545.52 (684,825.54–735,416.69)	1471.68 (1400.67–1504.14)	858,359.28 (768,975.00–943,619.13)	1382.69 (1238.70–1520.03)	−0.51 (−1.00 to −0.02)
High‐income Asia Pacific	1,615,115.45 (1,382,778.14–1,871,573.92)	4618.81 (3954.38–5352.21)	3,519,484.66 (2,997,552.88–4,135,939.65)	4991.94 (4251.64–5866.30)	0.03 (−0.10–0.16)	1,813,535.83 (1,681,705.71–1,889,189.54)	5186.24 (4809.24–5402.59)	2,050,794.60 (1,750,444.20–2,228,030.31)	2908.79 (2482.78–3160.18)	−1.87 (−2.03 to −1.72)	108,771.78 (98,245.26–113,995.78)	311.06 (280.96–326.00)	146,849.65 (118,204.85–163,008.68)	208.29 (167.66–231.21)	−1.27 (−1.48 to −1.05)
Australasia	504,165.62 (460,237.05–552,972.33)	12,797.67 (11,682.60–14,036.57)	957,561.99 (845,958.32–1,100,121.31)	10,839.20 (9575.89–12,452.91)	−0.79 (−0.97 to −0.61)	658,901.81 (619,800.55–679,417.01)	16,725.47 (15,732.93–17,246.23)	392,520.25 (347,230.54–419,227.70)	4443.16 (3930.50–4745.48)	−4.64 (−4.80 to −4.49)	38,042.78 (35,182.43–39,543.71)	965.67 (893.07–1003.77)	27,427.46 (23,208.55–29,744.33)	310.47 (262.71–336.69)	−4.03 (−4.15 to −3.91)
Western Europe	9,917,000.37 (8,731,907.53–11,160,072.11)	10,211.90 (8991.56–11,491.93)	11,976,445.80 (10,462,771.19–13,757,358.25)	8030.64 (7015.66–9224.80)	−0.94 (−1.01 to −0.88)	13,922,634.58 (13,140,921.53–14,341,040.67)	14,336.65 (13,531.69–14,767.49)	7,528,161.42 (6,662,923.38–8,017,444.59)	5047.90 (4467.73–5375.98)	−3.59 (−3.71 to −3.48)	837,249.48 (769,321.29–868,634.44)	862.15 (792.20–894.46)	526,365.68 (447,278.63–567,424.50)	352.95 (299.92–380.48)	−3.09 (−3.19 to −2.99)
Southern Latin America	712,494.10 (644,758.43–788,311.51)	8994.47 (8139.38–9951.58)	1,157,672.28 (1,037,942.97–1,309,362.61)	7866.71 (7053.11–8897.49)	−0.70 (−0.80 to −0.61)	1,024,318.45 (984,723.46–1,053,517.86)	12,930.91 (12,431.07–13,299.52)	773,434.62 (723,822.82–806,872.91)	5255.70 (4918.58–5482.93)	−2.68 (−2.78 to −2.57)	57,413.40 (54,509.69–59,387.94)	724.78 (688.13–749.71)	45,583.43 (41,527.39–48,111.16)	309.75 (282.19–326.93)	−2.46 (−2.56 to −2.37)
High‐income North America	8,933,357.32 (7,517,137.61–10,510,046.95)	15,421.46 (12,976.67–18,143.27)	8,763,626.68 (7,393,037.27–10,506,856.11)	7787.52 (6569.59–93,36.58)	−2.77 (−3.02 to −2.51)	10,121,985.55 (9,362,518.85–10,490,683.73)	17,473.37 (16,162.32–18,109.84)	8,191,184.37 (7,425,053.89–8,618,181.55)	7278.83 (6598.04–7658.27)	−3.33 (−3.55 to −3.12)	608,706.65 (544,384.58–639,286.25)	1050.80 (939.76–1103.59)	506,378.47 (440,000.25–543,035.47)	449.98 (390.99–482.55)	−3.29 (−3.51 to −3.07)
Caribbean	658,291.65 (597,084.77–727,031.85)	15,274.56 (13,854.35–16,869.56)	1,419,922.55 (1,268,916.17–1,598,576.20)	15,336.49 (13,705.48–17,266.11)	−0.03 (−0.10–0.04)	726,251.81 (693,587.69–754,766.40)	16,851.46 (16,093.54–17,513.09)	1,001,843.71 (889,753.82–1,128,956.59)	10,820.85 (9610.17–12,193.78)	−1.48 (−1.66 to −1.30)	39,693.59 (37,731.60–41,056.45)	921.02 (875.50–952.65)	55,028.44 (49,093.30–61,486.54)	594.36 (530.25–664.11)	−1.49 (−1.65 to −1.34)
Andean Latin America	317,904.72 (285,552.91–355,641.81)	9473.04 (8509.01–10,597.55)	1,058,749.10 (929,930.91–1,194,738.41)	10,687.49 (9387.14–12,060.23)	0.45 (0.41–0.49)	264,055.39 (241,276.49–290,360.66)	7868.42 (7189.65–8652.28)	508,922.93 (431,492.63–608,560.47)	5137.30 (4355.68–6143.08)	−1.54 (−1.93 to −1.15)	14,553.81 (13,237.55–15,872.26)	433.68 (394.46–472.97)	29,422.34 (25,097.50–35,104.99)	297.00 (253.35–354.37)	−1.31 (−1.70 to −0.91)
Central Latin America	1,682,755.94 (1,491,115.74–1,905,228.23)	12,400.46 (10,988.23–14,039.89)	5,318,790.11 (4,642,855.40–6,223,572.08)	12,436.89 (10,856.36–14,552.54)	−0.08 (−0.11 to −0.05)	1,427,418.96 (1,373,642.97–1,458,932.57)	10,518.84 (10,122.56–10,751.07)	3,831,143.57 (3,453,824.06–4,247,220.28)	8958.34 (8076.06–9931.25)	−0.75 (−0.95 to −0.54)	76,448.82 (72,527.73–78,426.95)	563.36 (534.47–577.94)	221,427.73 (197,309.78–244,728.84)	517.76 (461.37–572.25)	−0.42 (−0.61 to −0.22)
Tropical Latin America	1,321,024.12 (1,108,098.88–1,554,578.15)	8724.62 (7318.37–10,267.11)	4,144,652.68 (3,464,576.77–4,949,502.54)	9356.33 (7821.09–11,173.23)	0.23 (0.20–0.26)	1,801,771.84 (1,716,271.85–1,853,921.22)	11,899.68 (11,335.00–12,244.10)	2,789,144.21 (2,587,573.37–2,913,473.08)	6296.34 (5841.31–6577.01)	−2.02 (−2.08 to −1.97)	88,356.66 (82,816.19–91,268.22)	583.55 (546.95–602.77)	139,596.28 (126,642.92–147,215.80)	315.13 (285.89–332.33)	−1.90 (−1.95 to −1.84)
North Africa and Middle East	8,302,900.72 (7,703,316.43–9,013,490.15)	29,376.44 (27,255.05–31,890.57)	22,377,731.50 (20,252,164.54–25,032,633.94)	29,354.08 (26,565.86–32,836.66)	−0.08 (−0.12 to −0.03)	6,489,532.53 (6,036,486.08–7,040,913.04)	22,960.57 (21,357.65–24,911.41)	12,506,390.11 (11,116,329.51–13,959,839.30)	16,405.31 (14,581.89–18,311.87)	−1.17 (−1.23 to −1.11)	314,742.10 (291,461.22–341,861.46)	1113.59 (1031.22–1209.54)	646,864.42 (574,763.29–713,640.58)	848.53 (753.95–936.12)	−0.86 (−0.92 to −0.80)
South Asia	17,963,711.67 (15,286,274.90–21,097,276.36)	18,920.67 (16,100.60–22,221.16)	52,651,087.86 (43,693,204.92–63,401,984.94)	21,205.06 (17,597.30–25,534.95)	0.37 (0.35–0.40)	12,017,243.66 (10,894,521.37–13,078,261.28)	12,657.42 (11,474.89–13,774.96)	33,956,161.23 (31,075,856.04–36,753,272.12)	13,675.74 (12,515.70–14,802.26)	0.31 (0.21–0.41)	526,695.19 (473,100.88–575,036.21)	554.75 (498.30–605.67)	1,626,381.88 (1,490,038.44–1,763,123.38)	655.02 (600.11–710.09)	0.66 (0.56–0.76)
Central Sub‐Saharan Africa	366,275.58 (329,142.12–409,759.84)	9740.59 (8753.08–10,897.00)	825,382.33 (726,547.60–934,985.38)	9147.01 (8051.71–10,361.65)	−0.32 (−0.38 to −0.27)	447,920.86 (351,318.72–569,941.82)	11,911.84 (9342.84–15,156.81)	902,083.82 (701,024.94–1,153,297.41)	9997.03 (7768.86–12,781.01)	−0.79 (−0.87 to −0.71)	19,626.59 (15,532.37–24,789.70)	521.94 (413.06–659.25)	41,092.25 (32,095.14–52,095.75)	455.39 (355.68–577.33)	−0.65 (−0.75 to −0.55)
Eastern Sub‐Saharan Africa	1,165,274.58 (1,013,289.67–1,323,287.21)	9578.41 (8329.12–10,877.26)	2,717,529.82 (2,320,874.73–3,185,524.40)	10,050.93 (8583.88–11,781.84)	0.11 (0.09–0.13)	794,861.08 (705,595.82–903,548.68)	6533.66 (5799.91–7427.06)	1,721,837.29 (1,495,118.67–1,973,507.59)	6368.31 (5529.78–7299.13)	−0.28 (−0.38 to −0.19)	35,306.75 (31,265.59–40,230.54)	290.22 (257.00–330.69)	80,844.50 (70,160.02–92,566.26)	299.01 (259.49–342.36)	−0.08 (−0.18–0.03)
Southern Sub‐Saharan Africa	595,214.73 (510,829.96–694,260.73)	13,451.86 (11,544.76–15,690.30)	1,241,478.79 (1,040,360.23–1,498,686.10)	12,752.36 (10,686.49–15,394.37)	−0.34 (−0.42 to −0.27)	276,505.95 (234,510.77–309,574.77)	6249.04 (5299.95–6996.39)	672,900.53 (626,441.05–726,838.81)	6911.97 (6434.75–7466.02)	0.23 (−0.25–0.72)	14,166.01 (11,926.31–15,896.89)	320.15 (269.53–359.27)	33,548.20 (30,978.72–36,180.59)	344.60 (318.21–371.64)	0.14 (−0.32–0.61)
Western Sub‐Saharan Africa	1,608,084.22 (1,400,564.93–1,841,752.32)	11,139.79 (9702.23–12,758.50)	3,757,920.66 (3,222,918.28–4,402,081.07)	11,691.21 (10,026.77–13,695.25)	0.18 (0.14–0.22)	1,320,816.81 (1,137,755.35–1,532,311.01)	9149.79 (7881.65–10,614.89)	2,739,612.22 (2,365,469.66–3,156,390.07)	8523.17 (7359.18–9819.80)	−0.24 (−0.40 to −0.07)	65,583.05 (56,470.42–75,565.60)	454.32 (391.19–523.47)	138,277.81 (120,686.15–157,663.35)	430.19 (375.46–490.50)	−0.14 (−0.30–0.02)

Abbreviations: DALYs, disability‐adjusted life years; EAPC, estimated annual percentage change; SDI, socio‐demographic index.

**Figure 1:**
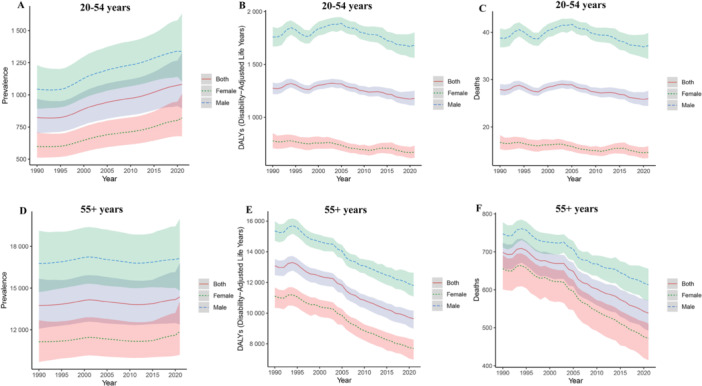
Global trends in IHD prevalence, DALY rates, and death rates, 1990–2021. (A) Prevalence in the 20–54‐year age group; (B) DALY rates in the 20–54‐year age group; (C) Death rates in the 20–54‐year age group; (D) Prevalence in the 55+ age group; (E) DALY rates in the 55+ age group; (F) Death rates in the 55+ age group. DALY, disability‐adjusted life year; IHD, ischemic heart disease.

From 1990 to 2021, IHD prevalence increased in both age groups, but the rise was markedly steeper among younger adults (EAPC = 0.98 [0.93–1.02]) than older adults (EAPC = 0.05 [0.01–0.09]) (Tables 1 and 2, Figure 1). Crucially, despite rising prevalence, mortality and DALY rates declined significantly in both groups. This decline was substantially greater in the older population (mortality EAPC = −0.93 [−1.00 to −0.86]; DALY EAPC = −1.12 [−1.19 to −1.05]) than in the younger population (mortality EAPC = −0.27 [−0.36 to −0.18]; DALY EAPC = −0.31 [−0.40 to −0.22]) (Tables 1 and 2, Figure 1).

Males consistently exhibited higher prevalence, mortality, and DALY rates than females across both age groups throughout the study period (Figure 1).

### 3.2 Comparative Analysis of Burden Across SDI Regions

**Figure 2:**
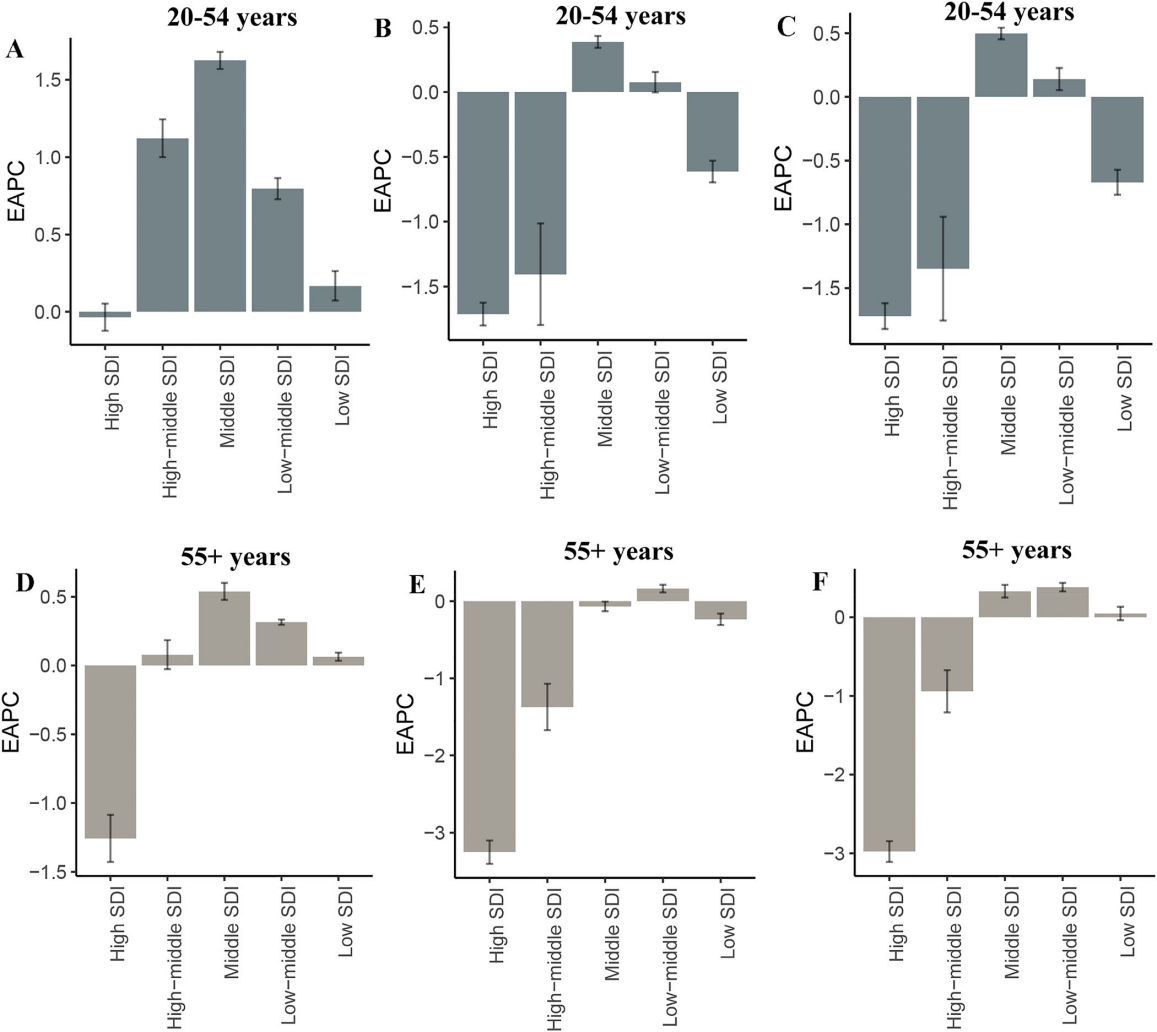
EAPC of IHD prevalence, DALY rates, and mortality rates across five SDI regions, 1990–2021. (A) EAPC of prevalence in the 20–54‐year age group; (B) EAPC of DALY rates in the 20–54‐year age group; (C) EAPC of mortality rates in the 20–54‐year age group; (D) EAPC of prevalence in the 55+ age group; (E) EAPC of DALY rates in the 55+ age group; (F) EAPC of mortality rates in the 55+ age group. DALY, disability‐adjusted life year; EAPC, estimated annual percentage change; IHD, ischemic heart disease.

In 2021, middle‐SDI regions globally carried the highest IHD burden among adults aged 20–54 years, demonstrating the highest prevalence, DALYs, and mortality across all SDI categories (Table 1). Temporal trends revealed divergent patterns by SDI level: while high‐SDI regions experienced a slight decline in prevalence (EAPC = −0.04 [−0.12 to 0.05]), all other SDI quintiles exhibited increasing prevalence, with the most pronounced rise observed in middle‐SDI regions (EAPC = 1.63 [1.57–1.68]). Conversely, mortality and DALY rates increased in middle‐ and low‐middle‐SDI regions but declined significantly in high‐SDI regions (Figure 2). Among adults aged ≥ 55 years, middle‐SDI regions similarly bore the greatest disease burden (Table 2). Notably, mortality and DALY rate trends in middle/low‐SDI regions showed opposing patterns compared to younger populations (Figure 2).

### 3.3 Comparative Analysis Across GBD Regions

**Figure 3:**
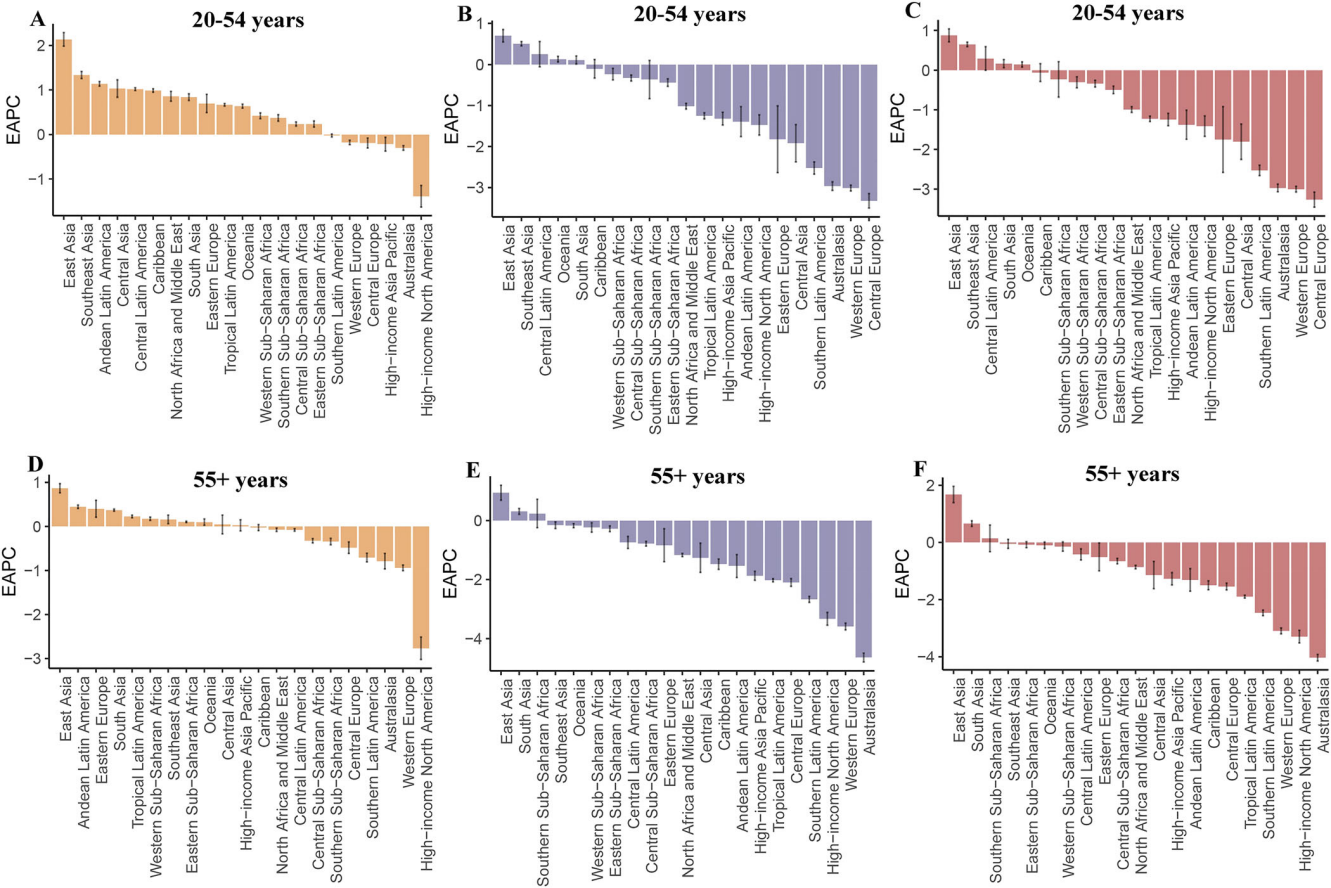
EAPC of IHD prevalence, DALY rates, and mortality rates across 21 GBD regions, 1990–2021. (A) EAPC of prevalence in the 20–54‐year age group; (B) EAPC of DALY rates in the 20–54‐year age group; (C) EAPC of mortality rates in the 20–54‐year age group; (D) EAPC of prevalence in the 55+ age group; (E) EAPC of DALY rates in the 55+ age group; (F) EAPC of mortality rates in the 55+ age group. DALY, disability‐adjusted life year; EAPC, estimated annual percentage change; GBD, global burden of disease; IHD, ischemic heart disease.

From 1990 to 2021, IHD prevalence increased across most regions in both age groups, with marked regional disparities. East Asia experienced the most pronounced growth among adults aged 20–54 years (EAPC = 2.14 [1.98–2.29]), followed by Southeast Asia (EAPC = 1.34 [1.25–1.42]). Similarly, in those aged ≥ 55 years, East Asia showed the largest increase (EAPC = 0.87 [0.76–0.98]). In sharp contrast, high‐income North America demonstrated significant declines in both age groups (20–54 years: EAPC = −1.39 [−1.63 to −1.15]; ≥ 55 years: EAPC = −2.77 [−3.02 to −2.51])—the steepest reductions globally (Tables 1 and 2, Figure 3).

While DALY rates decreased in most regions, East Asia exhibited concerning increases for both younger (EAPC = 0.70 [0.55–0.85]) and older adults (EAPC = 0.94 [0.69–1.20]). In addition, mortality rates attributable to IHD exhibited patterns similar to those of DALY rates across regions and age groups (Tables 1 and 2, Figure 3).

### 3.4 Comparative National Burden Analysis

Significant national disparities in IHD burden emerged in 2021. Among 20–54‐year‐olds, the United Arab Emirates (UAE) had the highest global prevalence (2785.63/100,000), while Brunei had the lowest (293.76/100,000). For ≥ 55‐year‐olds, Lebanon showed peak prevalence (36,227.41/100,000) while Brunei again had the minimum (3902.13/100,000) (Tables 1 and 2).

**Figure 4:**
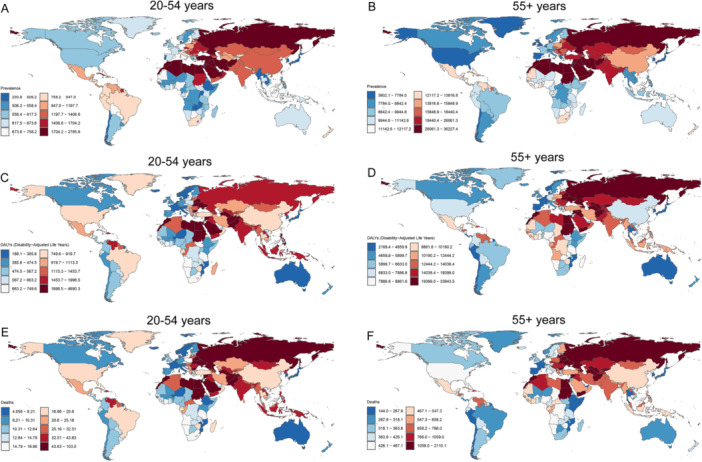
IHD prevalence, DALY rates, and mortality rates across 204 countries and territories in 2021. (A) Prevalence in the 20–54‐year age group; (B) Prevalence in the 55+ age group; (C) DALY rates in the 20–54‐year age group; (D) DALY rates in the 55+ age group; (E) Mortality rates in the 20–54‐year age group; (F) Mortality rates in the 55+ age group. DALY, disability‐adjusted life year; IHD, ischemic heart disease.

Temporally, Northern Mariana Islands led prevalence growth in younger adults (EAPC = 2.97 [2.50–3.44]), contrasting Finland's steepest decline (EAPC = −1.60 [−1.92 to 1.29]). Among older adults, Guam had maximal increase (EAPC = 1.27 [1.19–1.35]) opposed by the United States' sharpest reduction (EAPC = −2.88 [−3.14 to 2.61]) (Figure 4 and Supporting Information S1: Figure S1).

Notably, Nauru consistently bore the highest DALY burden across both age groups, with San Marino and South Korea exhibiting the respective lowest rates in younger and older populations. Mortality trends paralleled DALY patterns globally (Figure 4 and Supporting Information S1: Figure S1).

### 3.5 Correlation Analysis With the SDI

**Figure 5:**
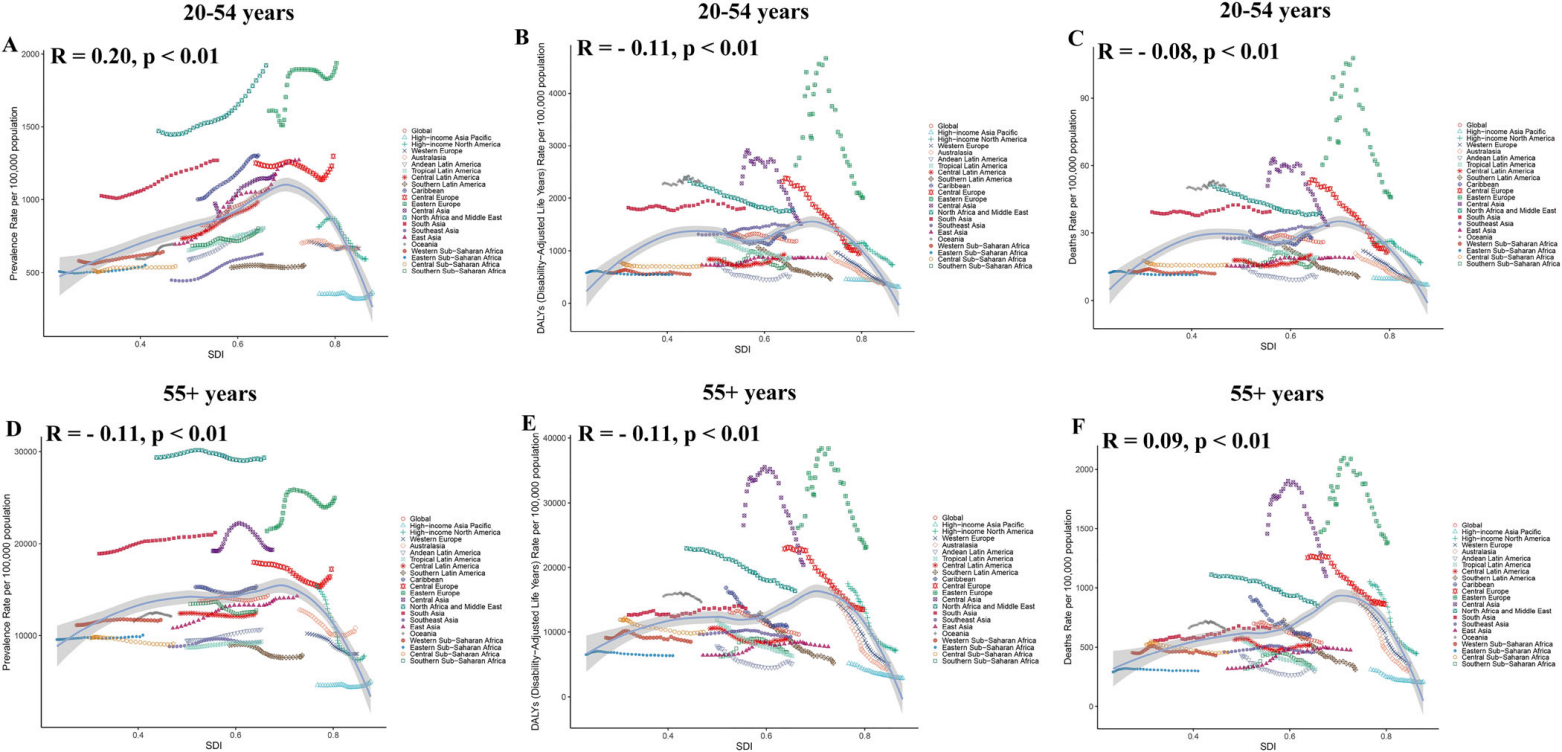
Correlation between SDI and IHD prevalence, DALY rates, and mortality rates in 2021 across 21 GBD regions. (A) Prevalence versus SDI in the 20–54‐year age group; (B) DALY rates versus SDI in the 20–54‐year age group; (C) Mortality rates versus SDI in the 20–54‐year age group; (D) Prevalence versus SDI in the 55+ age group; (E) DALY rates versus SDI in the 55+ age group; (F) Mortality rates versus SDI in the 55+ age group. DALY, disability‐adjusted life year; GBD, global burden of disease; IHD, ischemic heart disease; SDI, socio‐demographic index.

Correlation analyses revealed a paradoxical age‐dependent relationship between SDI and IHD metrics: among adults aged 20–54 years, prevalence increased with SDI (*R* = 0.20, *p* < 0.01) whereas mortality decreased (*R* = −0.08, *p* < 0.01); conversely, in those ≥ 55 years, prevalence declined with SDI (*R* = −0.11, *p* < 0.01) but mortality increased (*R* = 0.09, *p* < 0.01). Crucially, DALY rates exhibited consistent inverse correlations with SDI across both age groups (*R* = −0.11, *p* < 0.01 each) (Figure 5). Nation‐level analyses of 204 countries validated these regional patterns, though older adult mortality showed a marginally inverse correlation with SDI at finer geographic resolution (Supporting Information S1: Figure S2).

### 3.6 Comparative Analysis of Risk Factors

**Figure 6:**
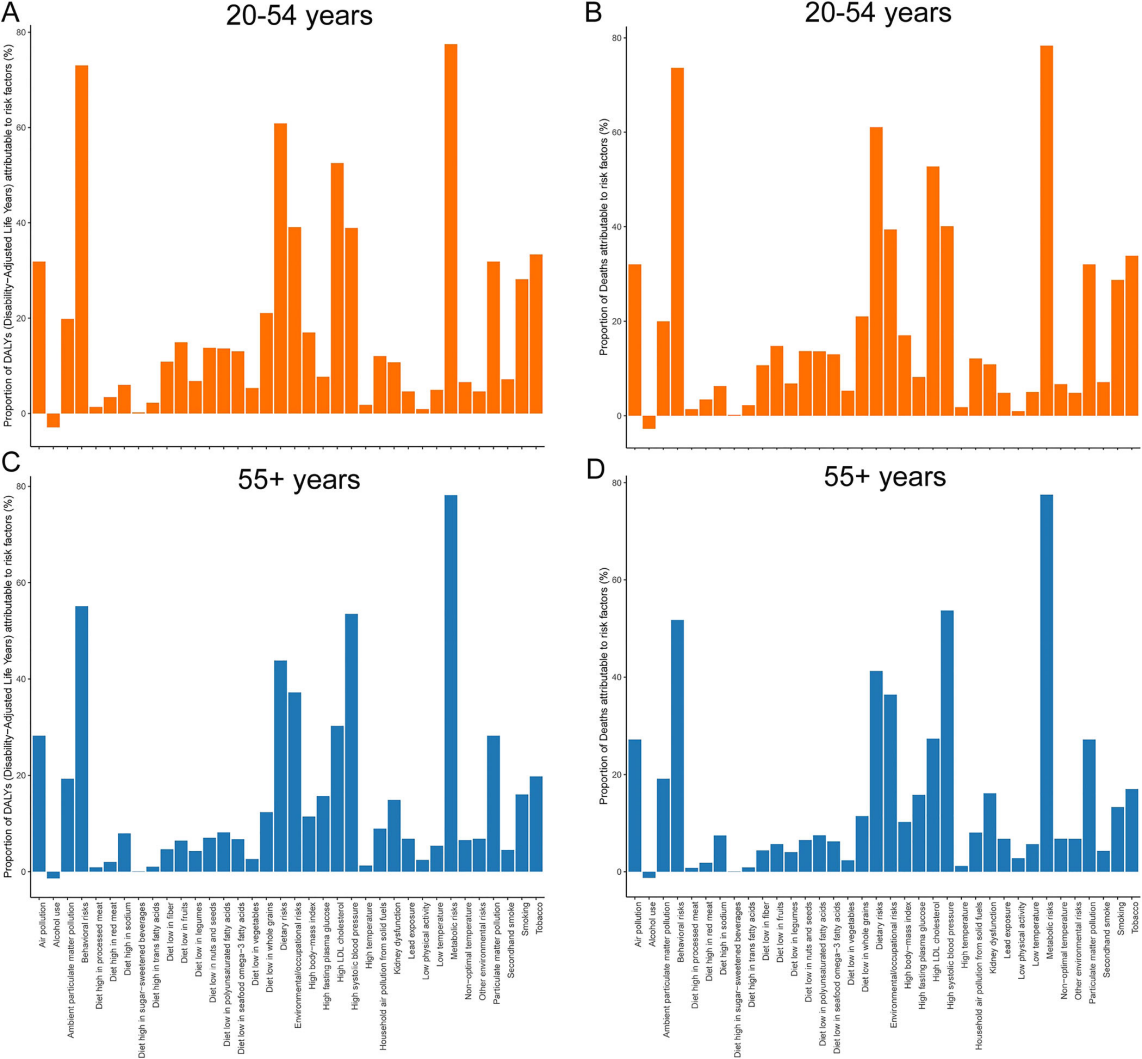
Global 2021 IHD DALYs and death proportions by risk factors across age groups. (A) DALYs in 20–54 age group; (B) Deaths in 20–54 age group; (C) DALYs in 55+ age group; (D) Deaths in 55+ age group. DALY, disability‐adjusted life year; IHD, ischemic heart disease.

The GBD 2021 identified 33 metabolic, environmental, and behavioral risk factors for IHD mortality and DALYs (Supporting Information S2: Table S1 and Supporting Information S3: Table S2). Age‐stratified analyses revealed that adults aged 20–54 years showed heightened susceptibility to tobacco use, high body mass index (BMI), elevated low‐density lipoprotein cholesterol (LDL‐C), and suboptimal diets, whereas those ≥ 55 years were predominantly affected by high blood pressure, elevated fasting plasma glucose, and renal dysfunction (Figure 6). Regionally, high‐SDI areas were characterized by high BMI, elevated fasting plasma glucose, and smoking dominance, contrasting with low‐SDI regions where ambient particulate matter pollution constituted the primary risk driver (Supporting Information S1: Figure S3A–D). Interestingly, we observed that alcohol consumption was inversely associated with the risk of IHD in both younger and older populations (Figure 6).

### 3.7 Forecasting Trends Using the BAPC Model

**Figure 7:**
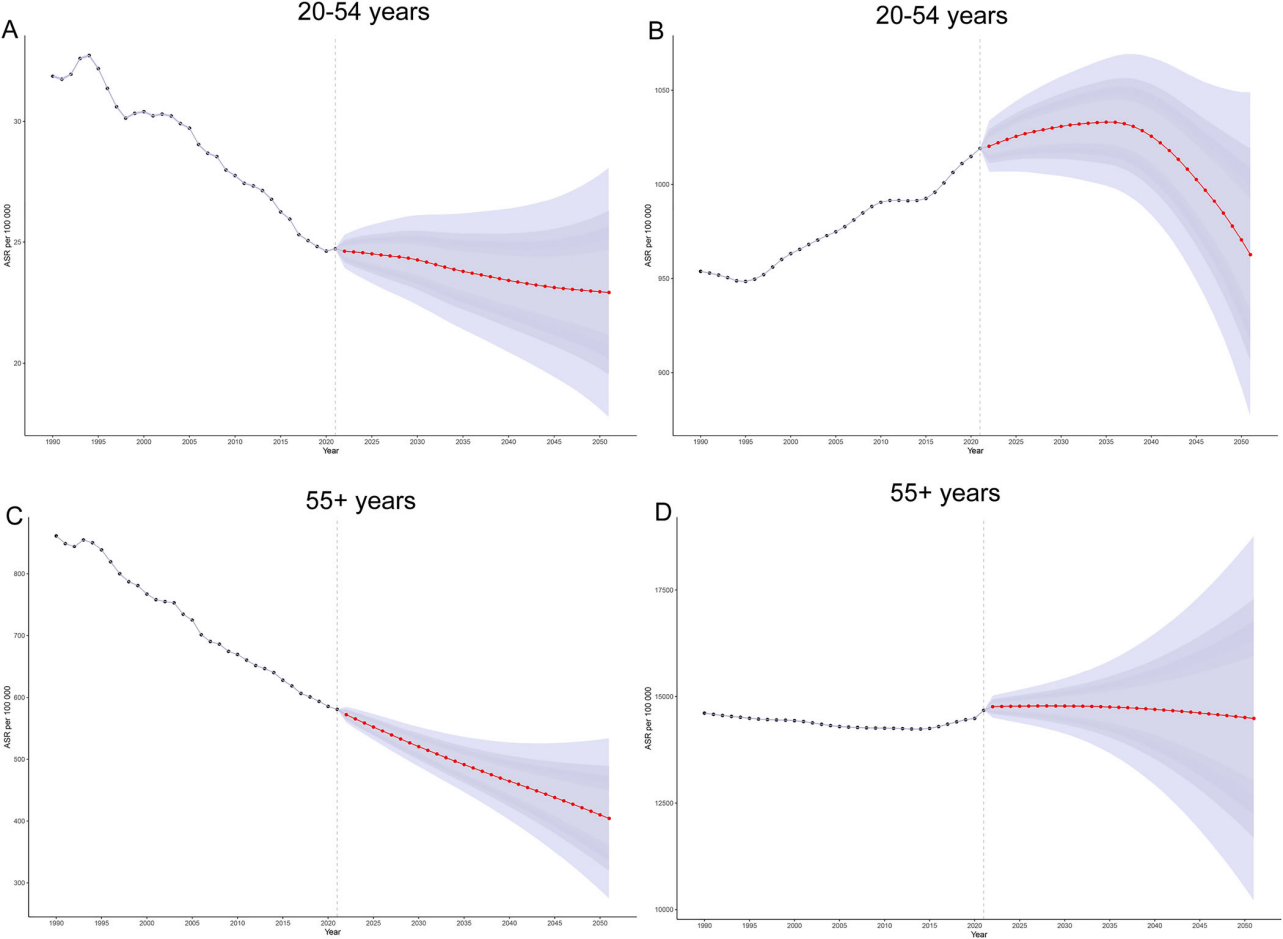
Projected global trends in IHD prevalence and death rates from 2021 to 2051 based on the BAPC model. (A) Death trends in the 20–54‐year age group; (B) Prevalence trends in the 20–54‐year age group; (C) Death trends in the 55+ age group; (D) Prevalence trends in the 55+ age group. ASR, age‐standardized rate; BAPC, Bayesian age‐period‐cohort; IHD, ischemic heart disease.

BAPC model projections indicate that over the next three decades, the mortality rate of IHD among the global population aged 20–54 will steadily decline, reaching approximately 22.92 per 100,000 by 2051, while the prevalence rate in this age group is expected to peak around 2035 at 1033.12 per 100,000 before gradually decreasing. For individuals aged 55 and older, mortality rates will continue their downward trend, hitting about 404.28 per 100,000 by 2051, with prevalence rates maintaining long‐term stability (Figure 7).

## 4 Discussion

Our study reveals three pivotal innovations in understanding global IHD burden: the rising prevalence contrasts sharply with the continuing decline in mortality across all age groups; regions with moderate SDI bear the highest absolute disease burden; metabolic and behavioral risk factors are the main drivers among young people, while environmental risk factors have a particularly significant impact in low‐SDI regions. These insights establish a novel, granular analytical framework for decoding the evolving dynamics of the global IHD crisis.

Recent GBD studies have illuminated the profound impact of population aging on IHD trends while projecting future cardiovascular mortality and DALYs through 2050 [13, 14]. However, these macro‐level analyses often overlook age‐specific dynamics, particularly for IHD subtypes. Our findings demonstrate that while mortality and DALYs rates for IHD have declined across all age groups, the prevalence of IHD in young adults aged 20–54 years has exhibited a marked surge since 1990. This increase significantly outpaces the rate observed in individuals aged ≥ 55 years. The reduction in mortality and DALYs rates is largely attributable to widespread adoption of evidence‐based medical practices, including standardized pharmacological therapies, acute myocardial infarction reperfusion techniques, and expanded secondary prevention programs [15, 16]. Conversely, the escalating prevalence among younger populations correlates with deteriorating metabolic risk profiles (e.g., obesity, diabetes, dyslipidemia), sedentary behavioral patterns, and increased psychosocial stressors‐trends that collectively underscore the epidemiological shift toward earlier disease onset [17, 18]. Notably, while age‐specific mortality rates have improved among older adults, the absolute disease burden continues to grow due to global demographic aging. Projections indicate that the proportion of individuals aged ≥ 65 years will double by 2050 [19], creating a dual burden: reduced per‐capita mortality risk coexists with expanding total case numbers driven by the growing elderly population. These findings highlight the urgency for tailored public health strategies: prioritizing primary prevention in younger cohorts through lifestyle interventions while strengthening geriatric care systems to mitigate the compounded effects of aging and comorbidities.

Our study revealed persistent gender disparities in IHD burden across age groups from 1990 to 2021 with males showing higher global prevalence mortality and DALY rates than females suggesting greater susceptibility potentially linked to behavioral factors like higher smoking alcohol consumption and occupational physical strain among men [20] and biological factors including premenopausal estrogen's protective effects on lipid homeostasis and endothelial function reducing women's atherosclerosis risk [21]. These differences highlight the need for sex‐specific prevention and management strategies.

The IHD burden reveals stark regional disparities across SDI quintiles, reflecting deep inequities in risk factor exposure and healthcare access. High‐SDI regions have achieved declines in prevalence, mortality, and DALYs through advanced preventive care, effective risk factor management, and widespread adoption of guideline‐directed therapies—including beta‐blockers, angiotensin‐converting enzyme inhibitors/angiotensin receptor blockers, and novel agents like sodium‐glucose cotransporter‐2 inhibitors—which have transformed heart failure outcomes [22]. Conversely, middle‐SDI regions bear the highest disease burden among younger adults, a trend linked to developmental challenges such as fragmented healthcare systems, workforce shortages, and limited access to modern pharmacological treatments [23]. Older populations in these regions face comparably severe outcomes, exacerbated by underinvestment in revascularization technologies like second‐generation drug‐eluting stents and hybrid procedures, which remain critical but underutilized tools for complex coronary disease [23]. Socioeconomic disparities worsen cardiovascular risks in low‐ and middle‐SDI regions where smoking, obesity, and hypertension hit younger populations hardest [24, 25]—older adults gain limited intervention benefits while younger groups lack prevention, perpetuating early disease and disability. Closing these gaps needs technology transfer from high‐SDI areas and policies targeting root health causes to harmonize progress and counter aging population impacts with persistent risks.

Across 21 GBD regions, the majority showed declines in mortality and DALYs, with Central Europe (younger) and Australasia (older) leading—a testament to high‐income regions' healthcare advancements [26]. Conversely, East Asia saw starkest rises in prevalence, especially among elders, fueled by aging demographics, prolonged lifespans, and lifestyle shifts, underscoring the urgency of tailored interventions for aging societies [27]. Nationally, the UAE, Lebanon, and Nauru ranked highest in IHD burden, while Brunei, San Marino, and South Korea held the lowest. Lebanon's elderly surge may be linked to conflict‐disrupted care [28]; the UAE's youth‐driven trends may be tied to urbanization and metabolic risks [29]. Such disparities highlight the necessity for country‐specific policies that address unique epidemiological, geopolitical, and socioeconomic drivers.

Our analysis highlights distinct risk factor profiles for IHD across age groups, driving disparities in disease burden. Among younger populations, high LDL‐C emerges as the dominant risk factor, contributing to over half of IHD‐related deaths (52.74%) and DALYs (52.56%), with 3.81 million cardiovascular deaths globally linked to elevated LDL‐C in 2021 [30]. This underscores the critical role of lipid management in mitigating young adult cardiovascular risk, aligning with evidence that LDL‐C control substantially reduces disease incidence [31]. In contrast, older populations face escalating burdens from high systolic blood pressure, which affected 4.06 billion individuals by 2019 and accounted for 76.65 million DALYs and 4.3 million deaths, exacerbated by comorbid diabetes and kidney disease [4]. Geographically, risk factor prevalence mirrors socioeconomic gradients: high‐SDI regions grapple with tobacco and diet‐driven risks, while low‐SDI areas confront environmental challenges like air pollution. Paradoxically, alcohol intake exhibited a consistent inverse association with IHD risk across ages, reflecting a potential “J‐shaped” dose–response pattern where moderate consumption may confer protection compared to abstention or heavy use [32, 33]. These findings emphasize the need for age‐ and context‐specific strategies to address modifiable risks, from lipid control in youth to hypertension management in aging societies, while leveraging regional insights to tailor interventions.

Our findings indicate global IHD mortality will decline for all age groups over the next 30 years due to medical advances and prevention efforts [34, 35, 36, 37], though younger populations will see initial prevalence increases until 2035 from unhealthy lifestyles that health education may reverse, while older groups will maintain stable prevalence as aging effects counterbalance healthcare gains [19]. Future age‐specific healthcare planning will be essential to manage IHD burdens.

This study has several limitations. First, it relies on multiple data sources, which may vary in quality and coverage, particularly in low‐ and middle‐income countries, potentially introducing biases. To mitigate this, we strictly adhered to GBD standardized data processing protocols and incorporated UIs for all estimates to quantify potential variability. Second, while the GBD database is regularly updated, it may not fully capture rapidly changing factors such as socioeconomic shifts or public health crises, including the COVID‐19 pandemic. We therefore limited our analysis to the 1990–2021 period to avoid pandemic‐related trend distortions. Third, the lack of detailed subcategorization of IHD (e.g., myocardial infarction, angina, or heart failure) in the GBD database limits the ability to distinguish acute from chronic presentations. As partial compensation, we will validate the consistency of this key trend through clinical literature in the future. Despite these limitations, our findings provide valuable insights into the global burden of IHD, emphasizing the need for adaptive public health strategies tailored to evolving demographic and regional characteristics.

## 5 Conclusions

Over the past three decades global IHD prevalence has risen across age groups while mortality and DALY rates declined with persistent disparities by age gender SDI region and country—future policies should prioritize age‐specific hypertension management lifestyle changes environmental improvements and targeted resource allocation in low‐middle‐income countries to reduce IHD burden and enhance global health outcomes.

## Author Contributions

**Yemin Wang:** writing – original draft, data curation, formal analysis. **Jing Li:** investigation, validation. **Hazrat Bilal:** conceptualization, software. **Xiao Yu:** writing – review and editing, funding acquisition. **Lei Sun:** writing – review and editing, supervision, resources. All authors have read and approved the final version of the manuscript.

## Conflicts of Interest

The authors declare no conflicts of interest.

## Transparency Statement

The lead authors, Xiao Yu and Lei Sun, affirm that this manuscript is an honest, accurate, and transparent account of the study being reported; that no important aspects of the study have been omitted; and that any discrepancies from the study as planned (and, if relevant, registered) have been explained.

## Supporting information

Supplementary File 2. **Figure S1:** EAPC of IHD prevalence, DALY rates, and death rates across 204 countries and territories, 1990‐2021. **Figure S2:** Correlation between SDI and IHD prevalence, DALY rates, and death rates in 2021 across 204 countries and territories. **Figure S3:** Proportion of IHD DALYs and deaths attributable to risk factors in the five SDI regions.

Supplementary File 2.

**Table S1:** Proportion of DALYs and deaths due to major risk factors in people aged 20‐54 years by SDI region, 2021.

**Table S2:** Proportion of DALYs and deaths due to major risk factors in people aged 55+ years by SDI region, 2021.

## Data Availability

The authors confirm that the data supporting the findings of this study are available within the article and its supplementary materials. Lei Sun and Xiao Yu had full access to all of the data in this study and takes complete responsibility for the integrity of the data and the accuracy of the data analysis.
