# Supplementary material for: The Rising Tide of Coronary Crisis: Decoding Age‐Specific Disparities in Ischemic Heart Disease Burden Through the Global Burden of Disease Study 2021 Revelations: An Ecological Study
Source: Health Sci Rep. 2025 Oct 15;8(10):e71244. doi: 10.1002/hsr2.71244 (PMC12528810; doi:10.1002/hsr2.71244)
Supplement: Supplementary file 1 — Supplementary File 2. Figure S1: EAPC of IHD prevalence, DALY rates, and death rates across 204 countries and territories, 1990‐2021. Figure S2: Correlation between SDI and IHD prevalence, DALY rates, and death rates in 2021 across 204 countries and territories. Figure S3: Proportion of IHD DALYs and deaths attributable to risk factors in the five SDI regions. [file HSR2-8-e71244-s001.docx]

**The Rising Tide of Coronary Crisis: Decoding Age-Specific Disparities in Ischemic Heart Disease Burden Through GBD 2021 Revelations**

**Supplementary Figures:**

**Figure S1.** EAPC of IHD prevalence, DALY rates, and death rates across 204 countries and territories, 1990-2021.

(A) EAPC of prevalence in the 20–54-year age group; (B) EAPC of prevalence in the 55+ age group; (C) EAPC of DALY rates in the 20–54-year age group; (D) EAPC of DALY rates in the 55+ age group; (E) EAPC of death rates in the 20–54-year age group; (F) EAPC of death rates in the 55+ age group. EAPC = estimated annual percentage change. IHD = ischemic heart disease. DALY = disability-adjusted life year.

**Figure S2.** Correlation between SDI and IHD prevalence, DALY rates, and death rates in 2021 across 204 countries and territories.

(A) Prevalence vs. SDI in the 20–54-year age group; (B) DALY rates vs. SDI in the 20–54-year age group; (C) Death rates vs. SDI in the 20–54-year age group; (D) Prevalence vs. SDI in the 55+ age group; (E) DALY rates vs. SDI in the 55+ age group; (F) Death rates vs. SDI in the 55+ age group. GBD = global burden of disease. SDI = socio-demographic index. IHD = ischemic heart disease. DALY = disability-adjusted life year.

**Figure S3.** Proportion of IHD DALYs and deaths attributable to risk factors in the five SDI regions.

(A) Proportion of DALYs in the 20–54-year age group; (B) Proportion of deaths in the 20–54-year age group; (C) Proportion of DALYs in the 55+ age group; (D) Proportion of deaths in the 55+ age group. IHD = ischemic heart disease. DALYs = disability-adjusted life years.


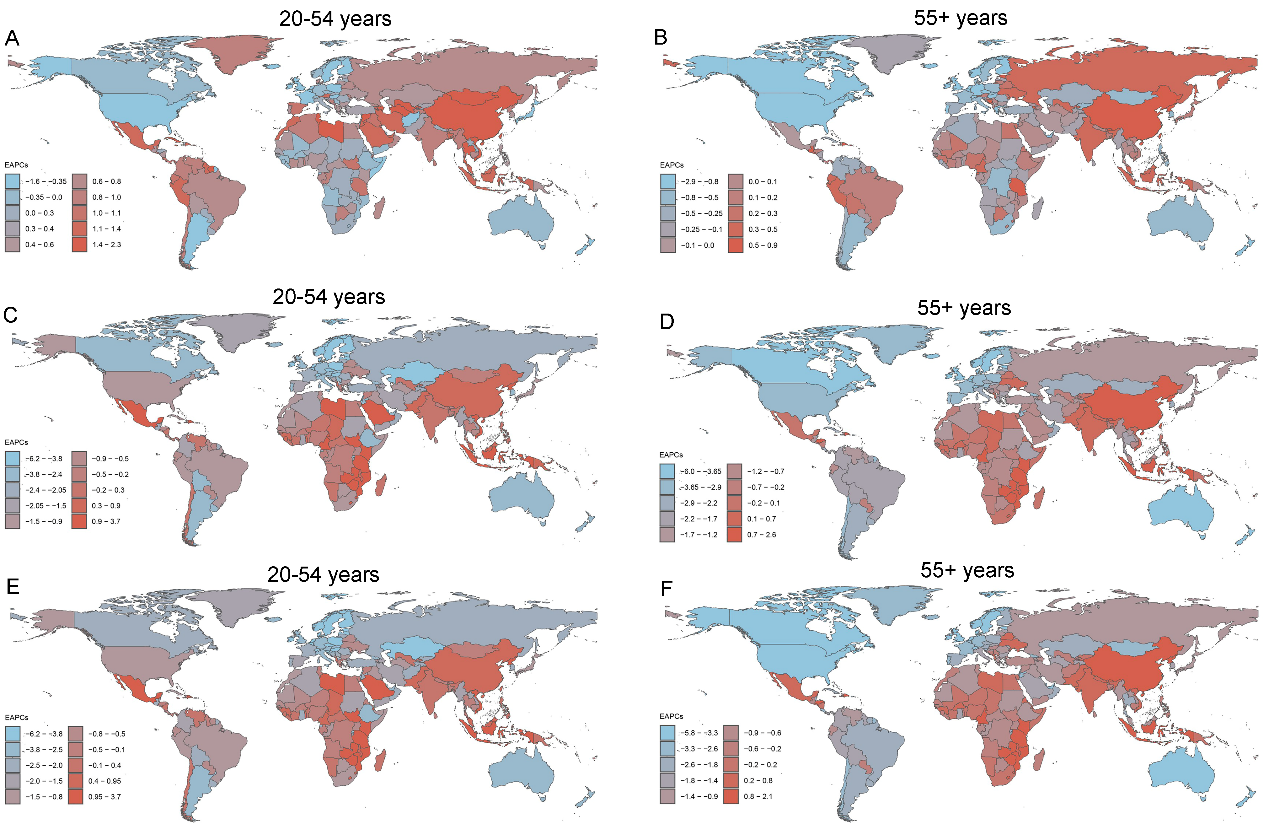


**Figure S1.** EAPC of IHD prevalence, DALY rates, and death rates across 204 countries and territories, 1990-2021.

(A) EAPC of prevalence in the 20–54-year age group; (B) EAPC of prevalence in the 55+ age group; (C) EAPC of DALY rates in the 20–54-year age group; (D) EAPC of DALY rates in the 55+ age group; (E) EAPC of death rates in the 20–54-year age group; (F) EAPC of death rates in the 55+ age group. EAPC = estimated annual percentage change. IHD = ischemic heart disease. DALY = disability-adjusted life year.


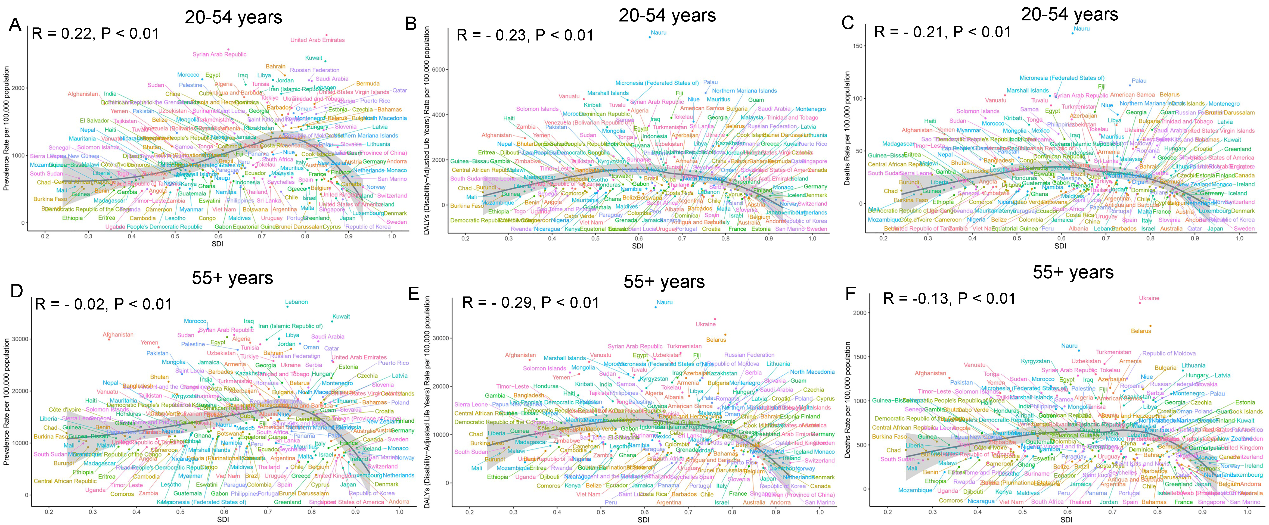


**Figure S2.** Correlation between SDI and IHD prevalence, DALY rates, and death rates in 2021 across 204 countries and territories.

(A) Prevalence vs. SDI in the 20–54-year age group; (B) DALY rates vs. SDI in the 20–54-year age group; (C) Death rates vs. SDI in the 20–54-year age group; (D) Prevalence vs. SDI in the 55+ age group; (E) DALY rates vs. SDI in the 55+ age group; (F) Death rates vs. SDI in the 55+ age group. GBD = global burden of disease. SDI = socio-demographic index. IHD = ischemic heart disease. DALY = disability-adjusted life year.


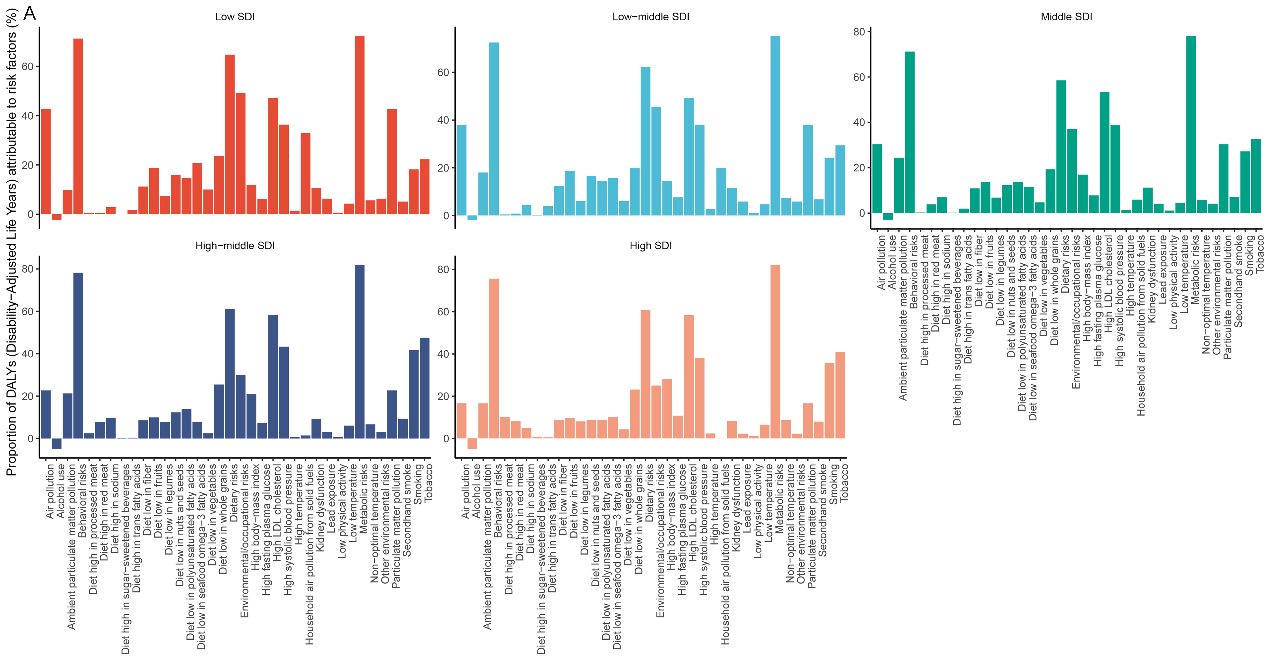


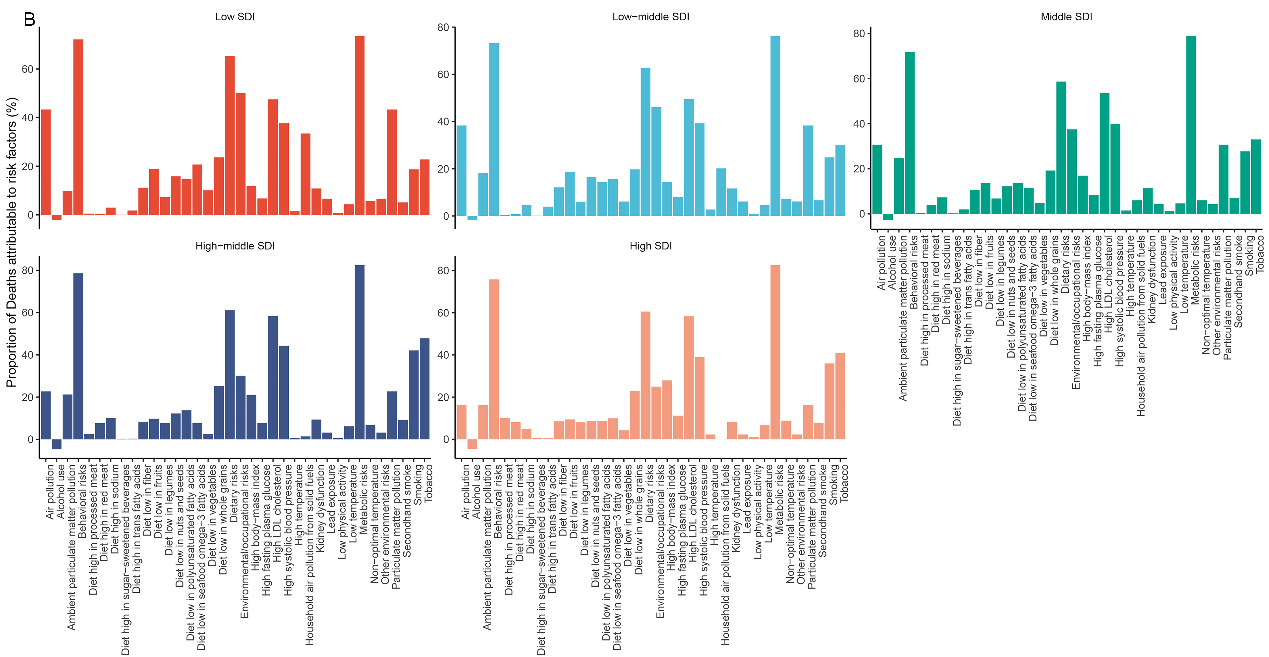


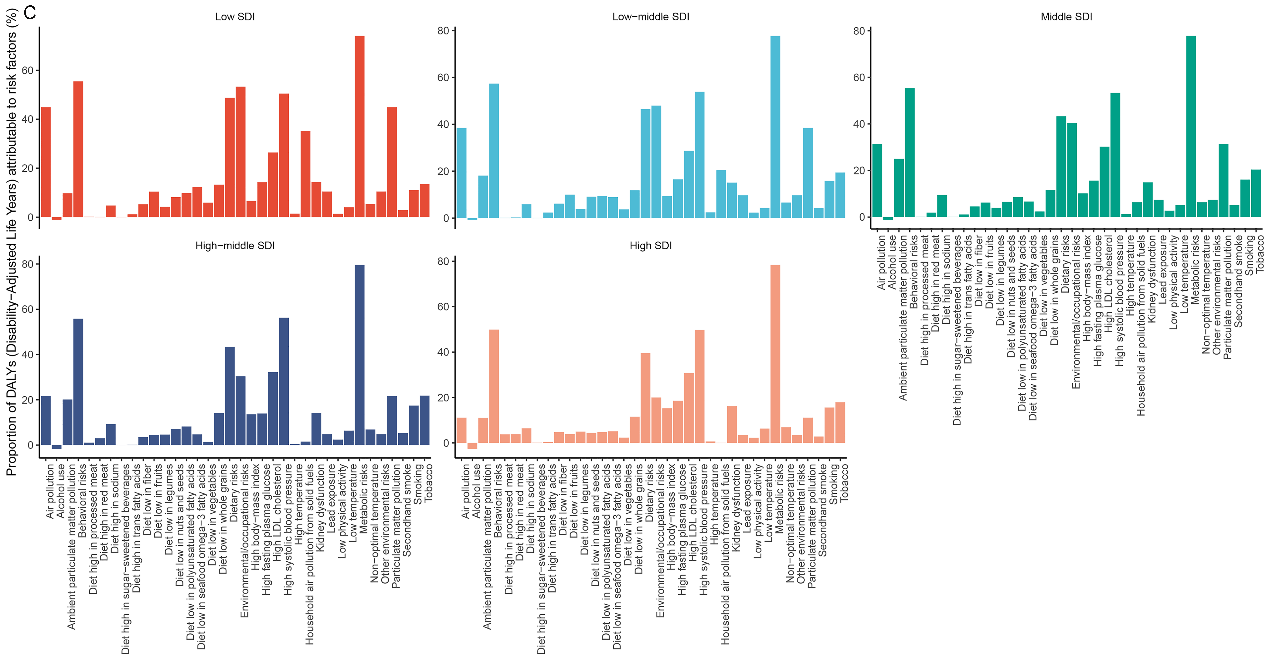


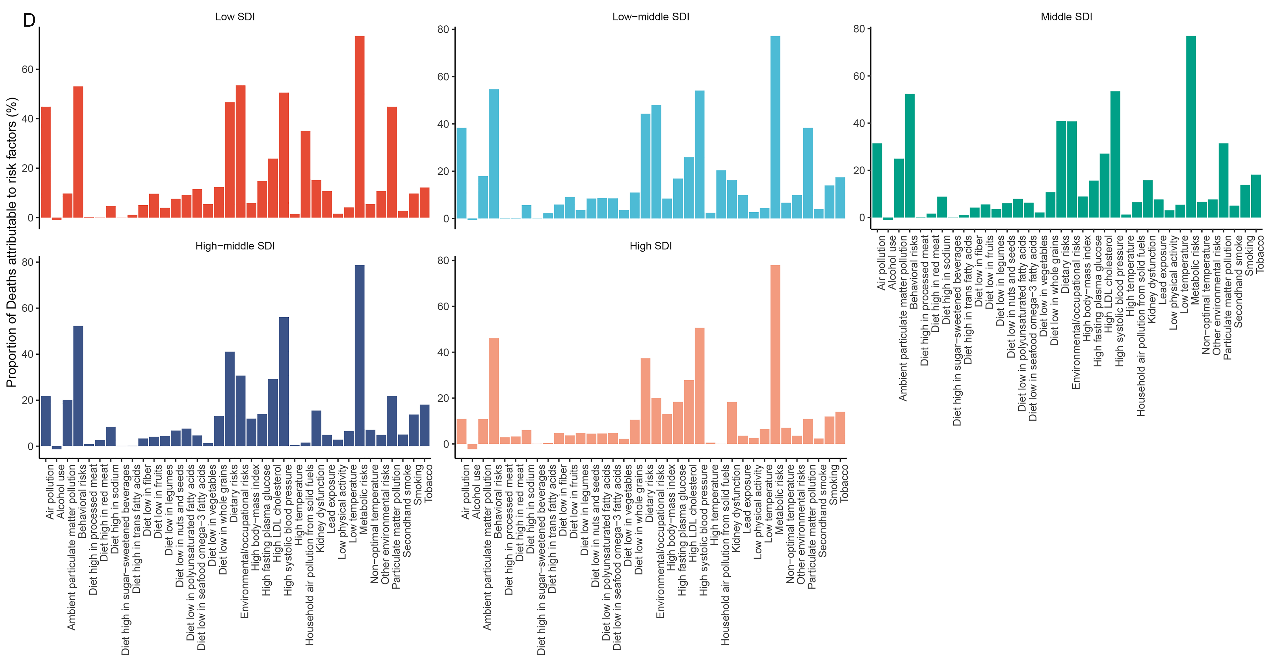


**Figure S3.** Proportion of IHD DALYs and deaths attributable to risk factors in the five SDI regions.

(A) Proportion of DALYs in the 20–54-year age group; (B) Proportion of deaths in the 20–54-year age group; (C) Proportion of DALYs in the 55+ age group; (D) Proportion of deaths in the 55+ age group. IHD = ischemic heart disease. DALYs = disability-adjusted life years.
