# Supplementary material for: The Rising Tide of Coronary Crisis: Decoding Age‐Specific Disparities in Ischemic Heart Disease Burden Through the Global Burden of Disease Study 2021 Revelations: An Ecological Study
Source: Health Sci Rep. 2025 Oct 15;8(10):e71244. doi: 10.1002/hsr2.71244 (PMC12528810; doi:10.1002/hsr2.71244)
Supplement: Supplementary file 2 [file HSR2-8-e71244-s003.docx]

**The Rising Tide of Coronary Crisis: Decoding Age-Specific Disparities in Ischemic Heart Disease Burden Through the Global Burden of Disease Study 2021 Revelations: An Ecological Study**

**2.1 Study population and data collection**

The GBD 2021 database encompasses epidemiological data for 371 diseases and injuries across 21 global regions, spanning 1990–2021^1^. This study focused on IHD-specific metrics, including age-standardized prevalence, mortality, and DALYs. Data extraction adhered to GBD protocols, ensuring consistency with global health benchmarks.

**2.2 Socio-demographic index**

The Socio-demographic Index (SDI) is a composite indicator ranging from 0.0 to 1.0, with 0.0 representing the lowest level of social and demographic development and 1.0 indicating the highest. It is calculated as the average of three standardized dimensions: (1) income level based on gross national income per capita, (2) education level measured by average years of schooling in adults aged ≥15 years, and (3) health status represented by birth-expectancy. This index stratifies regional development into three categories: low SDI (0.0-0.3) indicating foundational development needs, medium SDI (0.3-0.7) reflecting transitional growth phases, and high SDI (0.7-1.0) denoting advanced socioeconomic status. As a simplified assessment tool developed by the World Health Organization (WHO), SDI facilitates prioritized resource allocation and epidemiological analysis across diverse healthcare systems^1^.

**2.3 Bayesian Age-Period-Cohort (BAPC) analysis**

The BAPC model was implemented using R (version 4.4.1) with the “INLA” and “BAPC” packages. Model specifications included:

$Yap$ ~ Poisson($\mu_{ap}$)

$$\log\left( \mu_{ap} \right)=\mu+\alpha_{a}+\beta_{p}+\gamma_{c}$$

The observed event counts $Yap$for age group $a$ and period $p$ were modeled under a Poisson distribution, formulated as $Yap$ ~ Poisson($\mu_{ap}$). The log-linear predictor was specified by decomposing the log-expected count log($\mu_{ap}$​) into an intercept term $\mu$ (representing fixed effects) and random effects capturing age ($\alpha_{a}$), period ($\beta_{p}$), and cohort ($\gamma_{c}$) influences, with cohort defined as$c= p-a$ to account for temporal trends^2^.

**2.4 Statistical analysis (Supplementary Details)**

95% Uncertainty intervals (UIs) for prevalence, mortality, and DALYs were derived from 1,000 ordered estimates generated by GBD’s meta-regression framework. These intervals accounted for heterogeneity in data sources, statistical methods, and regional disparities. The EAPC formula was applied as:

$$y = \alpha+ \beta x + \varepsilon$$

EAPC = $100 \times（exp（\beta）- 1）$

where $x$ represents the year, $y$ denotes the natural logarithm of the rate (e.g., mortality rate), $\alpha$ denotes the intercept, $\beta$denotes the slope of the line, and $\varepsilon$ denotes the random error term. In previous studies, EAPC has been extensively utilized to monitor temporal trends in health metrics. A negative EAPC (<0) indicates an overall declining trend during the observation period, while a positive EAPC (>0) signifies an increasing trend. The magnitude of the absolute EAPC value reflects the steepness of the trend.

**References**

1. Global incidence, prevalence, years lived with disability (YLDs), disability-adjusted life-years (DALYs), and healthy life expectancy (HALE) for 371 diseases and injuries in 204 countries and territories and 811 subnational locations, 1990-2021: a systematic analysis for the Global Burden of Disease Study 2021. *Lancet (London, England).* 2024;403(10440):2133-2161.

2. Bai Z, Han J, An J, et al. The global, regional, and national patterns of change in the burden of congenital birth defects, 1990-2021: an analysis of the global burden of disease study 2021 and forecast to 2040. *EClinicalMedicine.* 2024;77:102873.
