# Supplementary material for: The Rising Tide of Coronary Crisis: Decoding Age‐Specific Disparities in Ischemic Heart Disease Burden Through the Global Burden of Disease Study 2021 Revelations: An Ecological Study
Source: Health Sci Rep. 2025 Oct 15;8(10):e71244. doi: 10.1002/hsr2.71244 (PMC12528810; doi:10.1002/hsr2.71244)
Supplement: Supplementary file 3 — Table S1: Proportion of DALYs and deaths due to major risk factors in people aged 20‐54 years by SDI region, 2021. [file HSR2-8-e71244-s002.docx]

**Table S1.** Proportion of DALYs and deaths due to major risk factors in people aged 20-54 years by SDI region, 2021.

| **Measure name** | **Location** | **Risk factor** | **Percent (%)** |
| --- | --- | --- | --- |
| DALYs | High SDI | Metabolic risks | 82.20 |
| DALYs | High-middle SDI | Metabolic risks | 81.94 |
| DALYs | High-middle SDI | Behavioral risks | 78.21 |
| DALYs | Middle SDI | Metabolic risks | 78.13 |
| DALYs | Global | Metabolic risks | 77.52 |
| DALYs | High SDI | Behavioral risks | 75.68 |
| DALYs | Low-middle SDI | Metabolic risks | 75.32 |
| DALYs | Global | Behavioral risks | 73.04 |
| DALYs | Low-middle SDI | Behavioral risks | 72.54 |
| DALYs | Low SDI | Metabolic risks | 72.40 |
| DALYs | Low SDI | Behavioral risks | 71.29 |
| DALYs | Middle SDI | Behavioral risks | 71.20 |
| DALYs | Low SDI | Dietary risks | 64.77 |
| DALYs | Low-middle SDI | Dietary risks | 62.28 |
| DALYs | High-middle SDI | Dietary risks | 61.04 |
| DALYs | Global | Dietary risks | 60.90 |
| DALYs | High SDI | Dietary risks | 60.74 |
| DALYs | Middle SDI | Dietary risks | 58.50 |
| DALYs | High SDI | High LDL cholesterol | 58.41 |
| DALYs | High-middle SDI | High LDL cholesterol | 58.41 |
| DALYs | Middle SDI | High LDL cholesterol | 53.41 |
| DALYs | Global | High LDL cholesterol | 52.56 |
| DALYs | Low-middle SDI | High LDL cholesterol | 49.29 |
| DALYs | Low SDI | Environmental/occupational risks | 49.25 |
| DALYs | High-middle SDI | Tobacco | 47.57 |
| DALYs | Low SDI | High LDL cholesterol | 47.13 |
| DALYs | Low-middle SDI | Environmental/occupational risks | 45.58 |
| DALYs | High-middle SDI | High systolic blood pressure | 43.33 |
| DALYs | Low SDI | Particulate matter pollution | 42.73 |
| DALYs | Low SDI | Air pollution | 42.73 |
| DALYs | High-middle SDI | Smoking | 41.76 |
| DALYs | High SDI | Tobacco | 40.96 |
| DALYs | Global | Environmental/occupational risks | 39.08 |
| DALYs | Global | High systolic blood pressure | 38.92 |
| DALYs | Middle SDI | High systolic blood pressure | 38.73 |
| DALYs | High SDI | High systolic blood pressure | 38.03 |
| DALYs | Low-middle SDI | High systolic blood pressure | 38.03 |
| DALYs | Low-middle SDI | Particulate matter pollution | 37.94 |
| DALYs | Low-middle SDI | Air pollution | 37.94 |
| DALYs | Middle SDI | Environmental/occupational risks | 36.99 |
| DALYs | Low SDI | High systolic blood pressure | 36.36 |
| DALYs | High SDI | Smoking | 35.81 |
| DALYs | Global | Tobacco | 33.41 |
| DALYs | Low SDI | Household air pollution from solid fuels | 32.98 |
| DALYs | Middle SDI | Tobacco | 32.54 |
| DALYs | Global | Particulate matter pollution | 31.91 |
| DALYs | Global | Air pollution | 31.91 |
| DALYs | Middle SDI | Particulate matter pollution | 30.37 |
| DALYs | Middle SDI | Air pollution | 30.37 |
| DALYs | High-middle SDI | Environmental/occupational risks | 29.93 |
| DALYs | Low-middle SDI | Tobacco | 29.42 |
| DALYs | Global | Smoking | 28.17 |
| DALYs | High SDI | High body-mass index | 28.01 |
| DALYs | Middle SDI | Smoking | 27.20 |
| DALYs | High-middle SDI | Diet low in whole grains | 25.45 |
| DALYs | High SDI | Environmental/occupational risks | 25.03 |
| DALYs | Middle SDI | Ambient particulate matter pollution | 24.39 |
| DALYs | Low-middle SDI | Smoking | 24.22 |
| DALYs | Low SDI | Diet low in whole grains | 23.62 |
| DALYs | High SDI | Diet low in whole grains | 23.15 |
| DALYs | High-middle SDI | Particulate matter pollution | 22.69 |
| DALYs | High-middle SDI | Air pollution | 22.69 |
| DALYs | Low SDI | Tobacco | 22.30 |
| DALYs | High-middle SDI | Ambient particulate matter pollution | 21.28 |
| DALYs | Global | Diet low in whole grains | 21.08 |
| DALYs | High-middle SDI | High body-mass index | 20.89 |
| DALYs | Low SDI | Diet low in seafood omega-3 fatty acids | 20.69 |
| DALYs | Low-middle SDI | Household air pollution from solid fuels | 19.90 |
| DALYs | Global | Ambient particulate matter pollution | 19.85 |
| DALYs | Low-middle SDI | Diet low in whole grains | 19.79 |
| DALYs | Middle SDI | Diet low in whole grains | 19.28 |
| DALYs | Low SDI | Diet low in fruits | 18.79 |
| DALYs | Low-middle SDI | Diet low in fruits | 18.61 |
| DALYs | Low SDI | Smoking | 18.19 |
| DALYs | Low-middle SDI | Ambient particulate matter pollution | 18.03 |
| DALYs | Global | High body-mass index | 17.00 |
| DALYs | Middle SDI | High body-mass index | 16.94 |
| DALYs | High SDI | Particulate matter pollution | 16.62 |
| DALYs | High SDI | Air pollution | 16.62 |
| DALYs | Low-middle SDI | Diet low in nuts and seeds | 16.61 |
| DALYs | High SDI | Ambient particulate matter pollution | 16.58 |
| DALYs | Low SDI | Diet low in nuts and seeds | 15.89 |
| DALYs | Low-middle SDI | Diet low in seafood omega-3 fatty acids | 15.66 |
| DALYs | Global | Diet low in fruits | 14.97 |
| DALYs | Low SDI | Diet low in polyunsaturated fatty acids | 14.71 |
| DALYs | Low-middle SDI | High body-mass index | 14.36 |
| DALYs | Low-middle SDI | Diet low in polyunsaturated fatty acids | 14.35 |
| DALYs | Global | Diet low in nuts and seeds | 13.83 |
| DALYs | High-middle SDI | Diet low in polyunsaturated fatty acids | 13.79 |
| DALYs | Middle SDI | Diet low in fruits | 13.76 |
| DALYs | Middle SDI | Diet low in polyunsaturated fatty acids | 13.74 |
| DALYs | Global | Diet low in polyunsaturated fatty acids | 13.68 |
| DALYs | Global | Diet low in seafood omega-3 fatty acids | 13.09 |
| DALYs | High-middle SDI | Diet low in nuts and seeds | 12.35 |
| DALYs | Middle SDI | Diet low in nuts and seeds | 12.32 |
| DALYs | Low-middle SDI | Diet low in fiber | 12.30 |
| DALYs | Global | Household air pollution from solid fuels | 12.05 |
| DALYs | Low SDI | High body-mass index | 11.84 |
| DALYs | Middle SDI | Diet low in seafood omega-3 fatty acids | 11.50 |
| DALYs | Low-middle SDI | Kidney dysfunction | 11.48 |
| DALYs | Low SDI | Diet low in fiber | 11.24 |
| DALYs | Middle SDI | Kidney dysfunction | 11.22 |
| DALYs | Global | Diet low in fiber | 10.88 |
| DALYs | Middle SDI | Diet low in fiber | 10.88 |
| DALYs | High SDI | High fasting plasma glucose | 10.76 |
| DALYs | Global | Kidney dysfunction | 10.75 |
| DALYs | Low SDI | Kidney dysfunction | 10.61 |
| DALYs | High SDI | Diet low in seafood omega-3 fatty acids | 10.06 |
| DALYs | High SDI | Diet high in processed meat | 10.04 |
| DALYs | Low SDI | Diet low in vegetables | 10.03 |
| DALYs | High-middle SDI | Diet low in fruits | 9.95 |
| DALYs | High-middle SDI | Diet high in sodium | 9.80 |
| DALYs | Low SDI | Ambient particulate matter pollution | 9.74 |
| DALYs | High SDI | Diet low in fruits | 9.62 |
| DALYs | High-middle SDI | Secondhand smoke | 9.31 |
| DALYs | High-middle SDI | Kidney dysfunction | 9.30 |
| DALYs | High SDI | Diet low in nuts and seeds | 8.76 |
| DALYs | High SDI | Non-optimal temperature | 8.68 |
| DALYs | High SDI | Diet low in polyunsaturated fatty acids | 8.68 |
| DALYs | High SDI | Diet low in fiber | 8.64 |
| DALYs | High-middle SDI | Diet low in fiber | 8.60 |
| DALYs | High SDI | Kidney dysfunction | 8.29 |
| DALYs | High SDI | Diet high in red meat | 8.21 |
| DALYs | High SDI | Diet low in legumes | 8.08 |
| DALYs | High SDI | Secondhand smoke | 7.95 |
| DALYs | High-middle SDI | Diet high in red meat | 7.82 |
| DALYs | Middle SDI | High fasting plasma glucose | 7.80 |
| DALYs | High-middle SDI | Diet low in seafood omega-3 fatty acids | 7.76 |
| DALYs | Global | High fasting plasma glucose | 7.73 |
| DALYs | High-middle SDI | Diet low in legumes | 7.66 |
| DALYs | Low-middle SDI | High fasting plasma glucose | 7.60 |
| DALYs | High-middle SDI | High fasting plasma glucose | 7.32 |
| DALYs | Low SDI | Diet low in legumes | 7.30 |
| DALYs | Low-middle SDI | Non-optimal temperature | 7.23 |
| DALYs | Global | Secondhand smoke | 7.20 |
| DALYs | Middle SDI | Secondhand smoke | 7.15 |
| DALYs | Middle SDI | Diet high in sodium | 6.97 |
| DALYs | Global | Diet low in legumes | 6.83 |
| DALYs | Middle SDI | Diet low in legumes | 6.82 |
| DALYs | Low-middle SDI | Secondhand smoke | 6.74 |
| DALYs | High-middle SDI | Non-optimal temperature | 6.67 |
| DALYs | Global | Non-optimal temperature | 6.62 |
| DALYs | High SDI | Low temperature | 6.51 |
| DALYs | Low SDI | Other environmental risks | 6.31 |
| DALYs | Low SDI | Lead exposure | 6.31 |
| DALYs | Low SDI | High fasting plasma glucose | 6.27 |
| DALYs | Low-middle SDI | Diet low in vegetables | 6.11 |
| DALYs | High-middle SDI | Low temperature | 6.08 |
| DALYs | Low-middle SDI | Diet low in legumes | 6.05 |
| DALYs | Global | Diet high in sodium | 6.04 |
| DALYs | Middle SDI | Household air pollution from solid fuels | 5.97 |
| DALYs | Middle SDI | Non-optimal temperature | 5.89 |
| DALYs | Low-middle SDI | Other environmental risks | 5.84 |
| DALYs | Low-middle SDI | Lead exposure | 5.84 |
| DALYs | Low SDI | Non-optimal temperature | 5.63 |
| DALYs | Global | Diet low in vegetables | 5.32 |
| DALYs | Low SDI | Secondhand smoke | 5.13 |
| DALYs | Global | Low temperature | 4.92 |
| DALYs | High SDI | Diet high in sodium | 4.91 |
| DALYs | Middle SDI | Diet low in vegetables | 4.74 |
| DALYs | Low-middle SDI | Low temperature | 4.65 |
| DALYs | Global | Other environmental risks | 4.61 |
| DALYs | Global | Lead exposure | 4.61 |
| DALYs | Middle SDI | Low temperature | 4.54 |
| DALYs | Low-middle SDI | Diet high in sodium | 4.52 |
| DALYs | Low SDI | Low temperature | 4.29 |
| DALYs | High SDI | Diet low in vegetables | 4.27 |
| DALYs | Middle SDI | Other environmental risks | 4.13 |
| DALYs | Middle SDI | Lead exposure | 4.13 |
| DALYs | Low-middle SDI | Diet high in trans fatty acids | 3.96 |
| DALYs | Middle SDI | Diet high in red meat | 3.85 |
| DALYs | Global | Diet high in red meat | 3.42 |
| DALYs | High-middle SDI | Other environmental risks | 3.08 |
| DALYs | High-middle SDI | Lead exposure | 3.08 |
| DALYs | Low SDI | Diet high in sodium | 2.85 |
| DALYs | Low-middle SDI | High temperature | 2.75 |
| DALYs | High-middle SDI | Diet high in processed meat | 2.46 |
| DALYs | High-middle SDI | Diet low in vegetables | 2.45 |
| DALYs | High SDI | High temperature | 2.34 |
| DALYs | Global | Diet high in trans fatty acids | 2.26 |
| DALYs | High SDI | Other environmental risks | 2.24 |
| DALYs | High SDI | Lead exposure | 2.24 |
| DALYs | Middle SDI | Diet high in trans fatty acids | 1.97 |
| DALYs | Global | High temperature | 1.81 |
| DALYs | Low SDI | Diet high in trans fatty acids | 1.76 |
| DALYs | Middle SDI | High temperature | 1.44 |
| DALYs | Low SDI | High temperature | 1.42 |
| DALYs | High-middle SDI | Household air pollution from solid fuels | 1.41 |
| DALYs | Global | Diet high in processed meat | 1.38 |
| DALYs | High SDI | Low physical activity | 1.15 |
| DALYs | Middle SDI | Low physical activity | 1.12 |
| DALYs | Global | Low physical activity | 0.95 |
| DALYs | Low-middle SDI | Low physical activity | 0.94 |
| DALYs | High SDI | Diet high in sugar-sweetened beverages | 0.8 |
| DALYs | Low-middle SDI | Diet high in red meat | 0.79 |
| DALYs | High-middle SDI | Low physical activity | 0.65 |
| DALYs | Low SDI | Low physical activity | 0.63 |
| DALYs | High-middle SDI | High temperature | 0.63 |
| DALYs | Low SDI | Diet high in processed meat | 0.52 |
| DALYs | High SDI | Diet high in trans fatty acids | 0.49 |
| DALYs | Low SDI | Diet high in red meat | 0.44 |
| DALYs | Low-middle SDI | Diet high in processed meat | 0.43 |
| DALYs | High-middle SDI | Diet high in trans fatty acids | 0.34 |
| DALYs | Middle SDI | Diet high in processed meat | 0.27 |
| DALYs | High-middle SDI | Diet high in sugar-sweetened beverages | 0.25 |
| DALYs | Global | Diet high in sugar-sweetened beverages | 0.23 |
| DALYs | Middle SDI | Diet high in sugar-sweetened beverages | 0.23 |
| DALYs | Low-middle SDI | Diet high in sugar-sweetened beverages | 0.14 |
| DALYs | Low SDI | Diet high in sugar-sweetened beverages | 0.09 |
| DALYs | High SDI | Household air pollution from solid fuels | 0.04 |
| DALYs | Low-middle SDI | Alcohol use | -1.82 |
| DALYs | Low SDI | Alcohol use | -2.26 |
| DALYs | Global | Alcohol use | -2.94 |
| DALYs | Middle SDI | Alcohol use | -2.95 |
| DALYs | High-middle SDI | Alcohol use | -4.86 |
| DALYs | High SDI | Alcohol use | -4.91 |
| Deaths | High SDI | Metabolic risks | 82.56 |
| Deaths | High-middle SDI | Metabolic risks | 82.47 |
| Deaths | Middle SDI | Metabolic risks | 78.96 |
| Deaths | High-middle SDI | Behavioral risks | 78.57 |
| Deaths | Global | Metabolic risks | 78.38 |
| Deaths | Low-middle SDI | Metabolic risks | 76.29 |
| Deaths | High SDI | Behavioral risks | 75.77 |
| Deaths | Global | Behavioral risks | 73.64 |
| Deaths | Low SDI | Metabolic risks | 73.61 |
| Deaths | Low-middle SDI | Behavioral risks | 73.23 |
| Deaths | Low SDI | Behavioral risks | 72.17 |
| Deaths | Middle SDI | Behavioral risks | 71.79 |
| Deaths | Low SDI | Dietary risks | 65.34 |
| Deaths | Low-middle SDI | Dietary risks | 62.62 |
| Deaths | Global | Dietary risks | 61.12 |
| Deaths | High-middle SDI | Dietary risks | 61.10 |
| Deaths | High SDI | Dietary risks | 60.57 |
| Deaths | Middle SDI | Dietary risks | 58.69 |
| Deaths | High-middle SDI | High LDL cholesterol | 58.44 |
| Deaths | High SDI | High LDL cholesterol | 58.29 |
| Deaths | Middle SDI | High LDL cholesterol | 53.53 |
| Deaths | Global | High LDL cholesterol | 52.74 |
| Deaths | Low SDI | Environmental/occupational risks | 50.04 |
| Deaths | Low-middle SDI | High LDL cholesterol | 49.49 |
| Deaths | High-middle SDI | Tobacco | 47.83 |
| Deaths | Low SDI | High LDL cholesterol | 47.50 |
| Deaths | Low-middle SDI | Environmental/occupational risks | 46.13 |
| Deaths | High-middle SDI | High systolic blood pressure | 44.30 |
| Deaths | Low SDI | Air pollution | 43.35 |
| Deaths | Low SDI | Particulate matter pollution | 43.35 |
| Deaths | High-middle SDI | Smoking | 42.11 |
| Deaths | High SDI | Tobacco | 41.05 |
| Deaths | Global | High systolic blood pressure | 40.11 |
| Deaths | Middle SDI | High systolic blood pressure | 39.98 |
| Deaths | Global | Environmental/occupational risks | 39.44 |
| Deaths | Low-middle SDI | High systolic blood pressure | 39.24 |
| Deaths | High SDI | High systolic blood pressure | 38.91 |
| Deaths | Low-middle SDI | Air pollution | 38.34 |
| Deaths | Low-middle SDI | Particulate matter pollution | 38.34 |
| Deaths | Low SDI | High systolic blood pressure | 37.64 |
| Deaths | Middle SDI | Environmental/occupational risks | 37.45 |
| Deaths | High SDI | Smoking | 35.99 |
| Deaths | Global | Tobacco | 33.90 |
| Deaths | Low SDI | Household air pollution from solid fuels | 33.45 |
| Deaths | Middle SDI | Tobacco | 32.99 |
| Deaths | Global | Particulate matter pollution | 32.09 |
| Deaths | Global | Air pollution | 32.09 |
| Deaths | Middle SDI | Air pollution | 30.63 |
| Deaths | Middle SDI | Particulate matter pollution | 30.63 |
| Deaths | High-middle SDI | Environmental/occupational risks | 30.11 |
| Deaths | Low-middle SDI | Tobacco | 29.94 |
| Deaths | Global | Smoking | 28.74 |
| Deaths | High SDI | High body-mass index | 27.94 |
| Deaths | Middle SDI | Smoking | 27.71 |
| Deaths | High-middle SDI | Diet low in whole grains | 25.31 |
| Deaths | High SDI | Environmental/occupational risks | 24.96 |
| Deaths | Low-middle SDI | Smoking | 24.83 |
| Deaths | Middle SDI | Ambient particulate matter pollution | 24.62 |
| Deaths | Low SDI | Diet low in whole grains | 23.66 |
| Deaths | High SDI | Diet low in whole grains | 22.92 |
| Deaths | Low SDI | Tobacco | 22.78 |
| Deaths | High-middle SDI | Air pollution | 22.69 |
| Deaths | High-middle SDI | Particulate matter pollution | 22.69 |
| Deaths | High-middle SDI | Ambient particulate matter pollution | 21.27 |
| Deaths | Global | Diet low in whole grains | 21.02 |
| Deaths | High-middle SDI | High body-mass index | 20.95 |
| Deaths | Low SDI | Diet low in seafood omega-3 fatty acids | 20.71 |
| Deaths | Low-middle SDI | Household air pollution from solid fuels | 20.14 |
| Deaths | Global | Ambient particulate matter pollution | 19.99 |
| Deaths | Low-middle SDI | Diet low in whole grains | 19.74 |
| Deaths | Middle SDI | Diet low in whole grains | 19.22 |
| Deaths | Low SDI | Diet low in fruits | 18.76 |
| Deaths | Low SDI | Smoking | 18.7 |
| Deaths | Low-middle SDI | Diet low in fruits | 18.55 |
| Deaths | Low-middle SDI | Ambient particulate matter pollution | 18.20 |
| Deaths | Global | High body-mass index | 17.04 |
| Deaths | Middle SDI | High body-mass index | 16.93 |
| Deaths | Low-middle SDI | Diet low in nuts and seeds | 16.56 |
| Deaths | High SDI | Air pollution | 16.34 |
| Deaths | High SDI | Particulate matter pollution | 16.34 |
| Deaths | High SDI | Ambient particulate matter pollution | 16.30 |
| Deaths | Low SDI | Diet low in nuts and seeds | 15.87 |
| Deaths | Low-middle SDI | Diet low in seafood omega-3 fatty acids | 15.57 |
| Deaths | Global | Diet low in fruits | 14.82 |
| Deaths | Low SDI | Diet low in polyunsaturated fatty acids | 14.81 |
| Deaths | Low-middle SDI | Diet low in polyunsaturated fatty acids | 14.39 |
| Deaths | Low-middle SDI | High body-mass index | 14.36 |
| Deaths | High-middle SDI | Diet low in polyunsaturated fatty acids | 13.75 |
| Deaths | Middle SDI | Diet low in polyunsaturated fatty acids | 13.74 |
| Deaths | Global | Diet low in nuts and seeds | 13.72 |
| Deaths | Global | Diet low in polyunsaturated fatty acids | 13.68 |
| Deaths | Middle SDI | Diet low in fruits | 13.60 |
| Deaths | Global | Diet low in seafood omega-3 fatty acids | 12.98 |
| Deaths | High-middle SDI | Diet low in nuts and seeds | 12.26 |
| Deaths | Middle SDI | Diet low in nuts and seeds | 12.21 |
| Deaths | Low-middle SDI | Diet low in fiber | 12.14 |
| Deaths | Global | Household air pollution from solid fuels | 12.10 |
| Deaths | Low SDI | High body-mass index | 11.93 |
| Deaths | Low-middle SDI | Kidney dysfunction | 11.66 |
| Deaths | Middle SDI | Diet low in seafood omega-3 fatty acids | 11.41 |
| Deaths | Middle SDI | Kidney dysfunction | 11.37 |
| Deaths | High SDI | High fasting plasma glucose | 11.21 |
| Deaths | Low SDI | Diet low in fiber | 11.14 |
| Deaths | Global | Kidney dysfunction | 10.88 |
| Deaths | Low SDI | Kidney dysfunction | 10.84 |
| Deaths | Global | Diet low in fiber | 10.68 |
| Deaths | Middle SDI | Diet low in fiber | 10.66 |
| Deaths | High-middle SDI | Diet high in sodium | 10.12 |
| Deaths | High SDI | Diet high in processed meat | 10.10 |
| Deaths | Low SDI | Diet low in vegetables | 10.07 |
| Deaths | High SDI | Diet low in seafood omega-3 fatty acids | 9.96 |
| Deaths | Low SDI | Ambient particulate matter pollution | 9.90 |
| Deaths | High-middle SDI | Diet low in fruits | 9.76 |
| Deaths | High SDI | Diet low in fruits | 9.43 |
| Deaths | High-middle SDI | Kidney dysfunction | 9.38 |
| Deaths | High-middle SDI | Secondhand smoke | 9.21 |
| Deaths | High SDI | Non-optimal temperature | 8.83 |
| Deaths | High SDI | Diet low in nuts and seeds | 8.56 |
| Deaths | High SDI | Diet low in polyunsaturated fatty acids | 8.54 |
| Deaths | High SDI | Diet low in fiber | 8.51 |
| Deaths | High-middle SDI | Diet low in fiber | 8.39 |
| Deaths | High SDI | Kidney dysfunction | 8.35 |
| Deaths | Middle SDI | High fasting plasma glucose | 8.29 |
| Deaths | Global | High fasting plasma glucose | 8.21 |
| Deaths | High SDI | Diet high in red meat | 8.19 |
| Deaths | Low-middle SDI | High fasting plasma glucose | 8.13 |
| Deaths | High SDI | Diet low in legumes | 8.06 |
| Deaths | High SDI | Secondhand smoke | 7.83 |
| Deaths | High-middle SDI | Diet high in red meat | 7.74 |
| Deaths | High-middle SDI | Diet low in seafood omega-3 fatty acids | 7.69 |
| Deaths | High-middle SDI | Diet low in legumes | 7.66 |
| Deaths | High-middle SDI | High fasting plasma glucose | 7.66 |
| Deaths | Low SDI | Diet low in legumes | 7.35 |
| Deaths | Middle SDI | Diet high in sodium | 7.30 |
| Deaths | Low-middle SDI | Non-optimal temperature | 7.26 |
| Deaths | Global | Secondhand smoke | 7.15 |
| Deaths | Middle SDI | Secondhand smoke | 7.1 |
| Deaths | Global | Diet low in legumes | 6.83 |
| Deaths | High-middle SDI | Non-optimal temperature | 6.81 |
| Deaths | Middle SDI | Diet low in legumes | 6.8 |
| Deaths | Low SDI | High fasting plasma glucose | 6.75 |
| Deaths | Global | Non-optimal temperature | 6.72 |
| Deaths | High SDI | Low temperature | 6.68 |
| Deaths | Low-middle SDI | Secondhand smoke | 6.66 |
| Deaths | Low SDI | Lead exposure | 6.63 |
| Deaths | Low SDI | Other environmental risks | 6.63 |
| Deaths | Global | Diet high in sodium | 6.32 |
| Deaths | High-middle SDI | Low temperature | 6.22 |
| Deaths | Low-middle SDI | Lead exposure | 6.14 |
| Deaths | Low-middle SDI | Other environmental risks | 6.14 |
| Deaths | Low-middle SDI | Diet low in vegetables | 6.11 |
| Deaths | Low-middle SDI | Diet low in legumes | 6.06 |
| Deaths | Middle SDI | Household air pollution from solid fuels | 6.01 |
| Deaths | Middle SDI | Non-optimal temperature | 6.00 |
| Deaths | Low SDI | Non-optimal temperature | 5.69 |
| Deaths | Global | Diet low in vegetables | 5.30 |
| Deaths | Low SDI | Secondhand smoke | 5.12 |
| Deaths | High SDI | Diet high in sodium | 5.03 |
| Deaths | Global | Low temperature | 5.01 |
| Deaths | Global | Other environmental risks | 4.82 |
| Deaths | Global | Lead exposure | 4.82 |
| Deaths | Low-middle SDI | Diet high in sodium | 4.73 |
| Deaths | Middle SDI | Diet low in vegetables | 4.72 |
| Deaths | Low-middle SDI | Low temperature | 4.67 |
| Deaths | Middle SDI | Low temperature | 4.63 |
| Deaths | Middle SDI | Lead exposure | 4.34 |
| Deaths | Middle SDI | Other environmental risks | 4.34 |
| Deaths | Low SDI | Low temperature | 4.33 |
| Deaths | High SDI | Diet low in vegetables | 4.24 |
| Deaths | Low-middle SDI | Diet high in trans fatty acids | 3.96 |
| Deaths | Middle SDI | Diet high in red meat | 3.82 |
| Deaths | Global | Diet high in red meat | 3.42 |
| Deaths | High-middle SDI | Lead exposure | 3.19 |
| Deaths | High-middle SDI | Other environmental risks | 3.19 |
| Deaths | Low SDI | Diet high in sodium | 3.00 |
| Deaths | Low-middle SDI | High temperature | 2.76 |
| Deaths | High-middle SDI | Diet high in processed meat | 2.47 |
| Deaths | High-middle SDI | Diet low in vegetables | 2.45 |
| Deaths | High SDI | Lead exposure | 2.32 |
| Deaths | High SDI | Other environmental risks | 2.32 |
| Deaths | High SDI | High temperature | 2.32 |
| Deaths | Global | Diet high in trans fatty acids | 2.24 |
| Deaths | Middle SDI | Diet high in trans fatty acids | 1.97 |
| Deaths | Global | High temperature | 1.82 |
| Deaths | Low SDI | Diet high in trans fatty acids | 1.77 |
| Deaths | Middle SDI | High temperature | 1.46 |
| Deaths | Low SDI | High temperature | 1.44 |
| Deaths | High-middle SDI | Household air pollution from solid fuels | 1.42 |
| Deaths | Global | Diet high in processed meat | 1.40 |
| Deaths | High SDI | Low physical activity | 1.17 |
| Deaths | Middle SDI | Low physical activity | 1.16 |
| Deaths | Global | Low physical activity | 0.98 |
| Deaths | Low-middle SDI | Low physical activity | 0.98 |
| Deaths | Low-middle SDI | Diet high in red meat | 0.77 |
| Deaths | High SDI | Diet high in sugar-sweetened beverages | 0.75 |
| Deaths | High-middle SDI | Low physical activity | 0.67 |
| Deaths | Low SDI | Low physical activity | 0.67 |
| Deaths | High-middle SDI | High temperature | 0.64 |
| Deaths | Low SDI | Diet high in processed meat | 0.51 |
| Deaths | High SDI | Diet high in trans fatty acids | 0.49 |
| Deaths | Low SDI | Diet high in red meat | 0.43 |
| Deaths | Low-middle SDI | Diet high in processed meat | 0.42 |
| Deaths | High-middle SDI | Diet high in trans fatty acids | 0.34 |
| Deaths | Middle SDI | Diet high in processed meat | 0.27 |
| Deaths | High-middle SDI | Diet high in sugar-sweetened beverages | 0.24 |
| Deaths | Global | Diet high in sugar-sweetened beverages | 0.22 |
| Deaths | Middle SDI | Diet high in sugar-sweetened beverages | 0.22 |
| Deaths | Low-middle SDI | Diet high in sugar-sweetened beverages | 0.14 |
| Deaths | Low SDI | Diet high in sugar-sweetened beverages | 0.08 |
| Deaths | High SDI | Household air pollution from solid fuels | 0.04 |
| Deaths | Low-middle SDI | Alcohol use | -1.71 |
| Deaths | Low SDI | Alcohol use | -2.12 |
| Deaths | Middle SDI | Alcohol use | -2.75 |
| Deaths | Global | Alcohol use | -2.79 |
| Deaths | High-middle SDI | Alcohol use | -4.65 |
| Deaths | High SDI | Alcohol use | -4.69 |

**Abbreviations:** SDI, socio-demographic index; DALYs, disability-adjusted life years.
