# Supplementary material for: The Rising Tide of Coronary Crisis: Decoding Age‐Specific Disparities in Ischemic Heart Disease Burden Through the Global Burden of Disease Study 2021 Revelations: An Ecological Study
Source: Health Sci Rep. 2025 Oct 15;8(10):e71244. doi: 10.1002/hsr2.71244 (PMC12528810; doi:10.1002/hsr2.71244)
Supplement: Supplementary file 4 — Table S2: Proportion of DALYs and deaths due to major risk factors in people aged 55+ years by SDI region, 2021. [file HSR2-8-e71244-s004.docx]

**Table S2.** Proportion of DALYs and deaths due to major risk factors in people aged 55+ years by SDI region, 2021.

| **Measure name** | **Location** | **Risk factor** | **Percent (%)** |
| --- | --- | --- | --- |
| DALYs | High-middle SDI | Metabolic risks | 79.62 |
| DALYs | High SDI | Metabolic risks | 78.41 |
| DALYs | Global | Metabolic risks | 78.14 |
| DALYs | Middle SDI | Metabolic risks | 77.82 |
| DALYs | Low-middle SDI | Metabolic risks | 77.74 |
| DALYs | Low SDI | Metabolic risks | 74.05 |
| DALYs | Low-middle SDI | Behavioral risks | 57.36 |
| DALYs | High-middle SDI | High systolic blood pressure | 56.24 |
| DALYs | High-middle SDI | Behavioral risks | 55.80 |
| DALYs | Low SDI | Behavioral risks | 55.47 |
| DALYs | Middle SDI | Behavioral risks | 55.38 |
| DALYs | Global | Behavioral risks | 55.15 |
| DALYs | Low-middle SDI | High systolic blood pressure | 53.88 |
| DALYs | Global | High systolic blood pressure | 53.54 |
| DALYs | Middle SDI | High systolic blood pressure | 53.31 |
| DALYs | Low SDI | Environmental/occupational risks | 53.28 |
| DALYs | Low SDI | High systolic blood pressure | 50.44 |
| DALYs | High SDI | Behavioral risks | 49.88 |
| DALYs | High SDI | High systolic blood pressure | 49.78 |
| DALYs | Low SDI | Dietary risks | 48.75 |
| DALYs | Low-middle SDI | Environmental/occupational risks | 47.97 |
| DALYs | Low-middle SDI | Dietary risks | 46.55 |
| DALYs | Low SDI | Air pollution | 44.86 |
| DALYs | Low SDI | Particulate matter pollution | 44.86 |
| DALYs | Global | Dietary risks | 43.83 |
| DALYs | High-middle SDI | Dietary risks | 43.40 |
| DALYs | Middle SDI | Dietary risks | 43.29 |
| DALYs | Middle SDI | Environmental/occupational risks | 40.35 |
| DALYs | High SDI | Dietary risks | 39.63 |
| DALYs | Low-middle SDI | Particulate matter pollution | 38.50 |
| DALYs | Low-middle SDI | Air pollution | 38.50 |
| DALYs | Global | Environmental/occupational risks | 37.23 |
| DALYs | Low SDI | Household air pollution from solid fuels | 35.11 |
| DALYs | High-middle SDI | High LDL cholesterol | 32.28 |
| DALYs | Middle SDI | Particulate matter pollution | 31.37 |
| DALYs | Middle SDI | Air pollution | 31.37 |
| DALYs | High SDI | High LDL cholesterol | 30.84 |
| DALYs | High-middle SDI | Environmental/occupational risks | 30.39 |
| DALYs | Global | High LDL cholesterol | 30.29 |
| DALYs | Middle SDI | High LDL cholesterol | 30.20 |
| DALYs | Low-middle SDI | High LDL cholesterol | 28.68 |
| DALYs | Global | Particulate matter pollution | 28.26 |
| DALYs | Global | Air pollution | 28.26 |
| DALYs | Low SDI | High LDL cholesterol | 26.40 |
| DALYs | Middle SDI | Ambient particulate matter pollution | 24.99 |
| DALYs | High-middle SDI | Tobacco | 21.84 |
| DALYs | High-middle SDI | Air pollution | 21.67 |
| DALYs | High-middle SDI | Particulate matter pollution | 21.67 |
| DALYs | Low-middle SDI | Household air pollution from solid fuels | 20.40 |
| DALYs | Middle SDI | Tobacco | 20.39 |
| DALYs | High-middle SDI | Ambient particulate matter pollution | 20.13 |
| DALYs | High SDI | Environmental/occupational risks | 20.01 |
| DALYs | Global | Tobacco | 19.81 |
| DALYs | Low-middle SDI | Tobacco | 19.44 |
| DALYs | Global | Ambient particulate matter pollution | 19.33 |
| DALYs | High SDI | High fasting plasma glucose | 18.57 |
| DALYs | Low-middle SDI | Ambient particulate matter pollution | 18.09 |
| DALYs | High SDI | Tobacco | 17.87 |
| DALYs | High-middle SDI | Smoking | 17.45 |
| DALYs | Low-middle SDI | High fasting plasma glucose | 16.51 |
| DALYs | High SDI | Kidney dysfunction | 16.18 |
| DALYs | Middle SDI | Smoking | 16.08 |
| DALYs | Global | Smoking | 16.03 |
| DALYs | Low-middle SDI | Smoking | 15.87 |
| DALYs | Global | High fasting plasma glucose | 15.70 |
| DALYs | Middle SDI | High fasting plasma glucose | 15.60 |
| DALYs | High SDI | Smoking | 15.59 |
| DALYs | High SDI | High body-mass index | 15.22 |
| DALYs | Low-middle SDI | Kidney dysfunction | 15.14 |
| DALYs | Global | Kidney dysfunction | 14.92 |
| DALYs | Middle SDI | Kidney dysfunction | 14.87 |
| DALYs | Low SDI | Kidney dysfunction | 14.38 |
| DALYs | Low SDI | High fasting plasma glucose | 14.26 |
| DALYs | High-middle SDI | Kidney dysfunction | 14.24 |
| DALYs | High-middle SDI | Diet low in whole grains | 14.14 |
| DALYs | High-middle SDI | High fasting plasma glucose | 13.93 |
| DALYs | High-middle SDI | High body-mass index | 13.59 |
| DALYs | Low SDI | Tobacco | 13.55 |
| DALYs | Low SDI | Diet low in whole grains | 13.27 |
| DALYs | Global | Diet low in whole grains | 12.39 |
| DALYs | Low SDI | Diet low in seafood omega-3 fatty acids | 12.23 |
| DALYs | Low-middle SDI | Diet low in whole grains | 11.83 |
| DALYs | High SDI | Diet low in whole grains | 11.55 |
| DALYs | Middle SDI | Diet low in whole grains | 11.54 |
| DALYs | Global | High body-mass index | 11.42 |
| DALYs | High SDI | Air pollution | 11.12 |
| DALYs | High SDI | Particulate matter pollution | 11.12 |
| DALYs | High SDI | Ambient particulate matter pollution | 11.06 |
| DALYs | Low SDI | Smoking | 11.03 |
| DALYs | Low SDI | Other environmental risks | 10.46 |
| DALYs | Low SDI | Lead exposure | 10.46 |
| DALYs | Low SDI | Diet low in fruits | 10.44 |
| DALYs | Middle SDI | High body-mass index | 10.17 |
| DALYs | Low-middle SDI | Diet low in fruits | 9.99 |
| DALYs | Low SDI | Diet low in polyunsaturated fatty acids | 9.79 |
| DALYs | Low SDI | Ambient particulate matter pollution | 9.75 |
| DALYs | Low-middle SDI | Other environmental risks | 9.71 |
| DALYs | Low-middle SDI | Lead exposure | 9.71 |
| DALYs | Middle SDI | Diet high in sodium | 9.48 |
| DALYs | Low-middle SDI | High body-mass index | 9.40 |
| DALYs | High-middle SDI | Diet high in sodium | 9.35 |
| DALYs | Low-middle SDI | Diet low in polyunsaturated fatty acids | 9.34 |
| DALYs | Low-middle SDI | Diet low in nuts and seeds | 9.12 |
| DALYs | Low-middle SDI | Diet low in seafood omega-3 fatty acids | 8.99 |
| DALYs | Global | Household air pollution from solid fuels | 8.93 |
| DALYs | Middle SDI | Diet low in polyunsaturated fatty acids | 8.56 |
| DALYs | High-middle SDI | Diet low in polyunsaturated fatty acids | 8.22 |
| DALYs | Low SDI | Diet low in nuts and seeds | 8.20 |
| DALYs | Global | Diet low in polyunsaturated fatty acids | 8.17 |
| DALYs | Global | Diet high in sodium | 7.96 |
| DALYs | Middle SDI | Other environmental risks | 7.40 |
| DALYs | Middle SDI | Lead exposure | 7.40 |
| DALYs | High-middle SDI | Diet low in nuts and seeds | 7.17 |
| DALYs | Global | Diet low in nuts and seeds | 7.05 |
| DALYs | High SDI | Non-optimal temperature | 6.90 |
| DALYs | High-middle SDI | Non-optimal temperature | 6.90 |
| DALYs | Global | Other environmental risks | 6.84 |
| DALYs | Global | Lead exposure | 6.84 |
| DALYs | Global | Diet low in seafood omega-3 fatty acids | 6.76 |
| DALYs | Middle SDI | Diet low in seafood omega-3 fatty acids | 6.66 |
| DALYs | Low SDI | High body-mass index | 6.64 |
| DALYs | Low-middle SDI | Non-optimal temperature | 6.62 |
| DALYs | Global | Non-optimal temperature | 6.58 |
| DALYs | Middle SDI | Diet low in nuts and seeds | 6.51 |
| DALYs | Global | Diet low in fruits | 6.47 |
| DALYs | High SDI | Diet high in sodium | 6.43 |
| DALYs | High-middle SDI | Low temperature | 6.41 |
| DALYs | Middle SDI | Non-optimal temperature | 6.37 |
| DALYs | Middle SDI | Household air pollution from solid fuels | 6.37 |
| DALYs | High SDI | Low temperature | 6.31 |
| DALYs | Middle SDI | Diet low in fruits | 6.21 |
| DALYs | Low-middle SDI | Diet low in fiber | 6.15 |
| DALYs | Low-middle SDI | Diet high in sodium | 5.90 |
| DALYs | Low SDI | Diet low in vegetables | 5.89 |
| DALYs | Global | Low temperature | 5.40 |
| DALYs | Low SDI | Non-optimal temperature | 5.39 |
| DALYs | High-middle SDI | Secondhand smoke | 5.35 |
| DALYs | Low SDI | Diet low in fiber | 5.20 |
| DALYs | Middle SDI | Low temperature | 5.15 |
| DALYs | Middle SDI | Secondhand smoke | 5.12 |
| DALYs | High SDI | Diet low in seafood omega-3 fatty acids | 5.09 |
| DALYs | High SDI | Diet low in legumes | 5.00 |
| DALYs | High-middle SDI | Other environmental risks | 4.80 |
| DALYs | High-middle SDI | Lead exposure | 4.80 |
| DALYs | High SDI | Diet low in fiber | 4.76 |
| DALYs | Low SDI | Diet high in sodium | 4.74 |
| DALYs | High SDI | Diet low in polyunsaturated fatty acids | 4.73 |
| DALYs | High-middle SDI | Diet low in seafood omega-3 fatty acids | 4.72 |
| DALYs | Global | Diet low in fiber | 4.68 |
| DALYs | High-middle SDI | Diet low in legumes | 4.64 |
| DALYs | Global | Secondhand smoke | 4.54 |
| DALYs | Middle SDI | Diet low in fiber | 4.54 |
| DALYs | High SDI | Diet low in nuts and seeds | 4.41 |
| DALYs | High-middle SDI | Diet low in fruits | 4.36 |
| DALYs | Low-middle SDI | Low temperature | 4.32 |
| DALYs | Global | Diet low in legumes | 4.27 |
| DALYs | Low-middle SDI | Secondhand smoke | 4.27 |
| DALYs | Low SDI | Diet low in legumes | 4.22 |
| DALYs | Low SDI | Low temperature | 4.03 |
| DALYs | High SDI | Diet low in fruits | 3.94 |
| DALYs | Middle SDI | Diet low in legumes | 3.92 |
| DALYs | Low-middle SDI | Diet low in legumes | 3.84 |
| DALYs | High SDI | Diet high in red meat | 3.83 |
| DALYs | High SDI | Diet high in processed meat | 3.79 |
| DALYs | Low-middle SDI | Diet low in vegetables | 3.71 |
| DALYs | High SDI | Other environmental risks | 3.51 |
| DALYs | High SDI | Lead exposure | 3.51 |
| DALYs | High-middle SDI | Diet low in fiber | 3.47 |
| DALYs | High-middle SDI | Diet high in red meat | 3.03 |
| DALYs | Low SDI | Secondhand smoke | 2.92 |
| DALYs | High SDI | Secondhand smoke | 2.82 |
| DALYs | Middle SDI | Low physical activity | 2.74 |
| DALYs | Global | Diet low in vegetables | 2.62 |
| DALYs | Global | Low physical activity | 2.43 |
| DALYs | Low-middle SDI | High temperature | 2.43 |
| DALYs | High-middle SDI | Low physical activity | 2.42 |
| DALYs | Low-middle SDI | Diet high in trans fatty acids | 2.38 |
| DALYs | Middle SDI | Diet low in vegetables | 2.38 |
| DALYs | High SDI | Diet low in vegetables | 2.35 |
| DALYs | Low-middle SDI | Low physical activity | 2.35 |
| DALYs | High SDI | Low physical activity | 2.28 |
| DALYs | Global | Diet high in red meat | 2.02 |
| DALYs | Middle SDI | Diet high in red meat | 1.91 |
| DALYs | High-middle SDI | Household air pollution from solid fuels | 1.54 |
| DALYs | Low SDI | High temperature | 1.44 |
| DALYs | High-middle SDI | Diet low in vegetables | 1.41 |
| DALYs | Low SDI | Low physical activity | 1.38 |
| DALYs | Middle SDI | High temperature | 1.31 |
| DALYs | Global | High temperature | 1.26 |
| DALYs | Low SDI | Diet high in trans fatty acids | 1.16 |
| DALYs | Middle SDI | Diet high in trans fatty acids | 1.10 |
| DALYs | High-middle SDI | Diet high in processed meat | 1.08 |
| DALYs | Global | Diet high in trans fatty acids | 1.04 |
| DALYs | Global | Diet high in processed meat | 0.90 |
| DALYs | High SDI | High temperature | 0.64 |
| DALYs | High-middle SDI | High temperature | 0.53 |
| DALYs | High SDI | Diet high in trans fatty acids | 0.41 |
| DALYs | Low-middle SDI | Diet high in red meat | 0.28 |
| DALYs | High-middle SDI | Diet high in trans fatty acids | 0.17 |
| DALYs | Low SDI | Diet high in processed meat | 0.17 |
| DALYs | High SDI | Diet high in sugar-sweetened beverages | 0.13 |
| DALYs | Low-middle SDI | Diet high in processed meat | 0.13 |
| DALYs | Middle SDI | Diet high in processed meat | 0.10 |
| DALYs | Global | Diet high in sugar-sweetened beverages | 0.07 |
| DALYs | High SDI | Household air pollution from solid fuels | 0.06 |
| DALYs | High-middle SDI | Diet high in sugar-sweetened beverages | 0.06 |
| DALYs | Low SDI | Diet high in red meat | 0.06 |
| DALYs | Middle SDI | Diet high in sugar-sweetened beverages | 0.06 |
| DALYs | Low-middle SDI | Diet high in sugar-sweetened beverages | 0.05 |
| DALYs | Low SDI | Diet high in sugar-sweetened beverages | 0.03 |
| DALYs | Low-middle SDI | Alcohol use | -0.73 |
| DALYs | Low SDI | Alcohol use | -1.08 |
| DALYs | Middle SDI | Alcohol use | -1.18 |
| DALYs | Global | Alcohol use | -1.39 |
| DALYs | High-middle SDI | Alcohol use | -1.62 |
| DALYs | High SDI | Alcohol use | -2.57 |
| Deaths | High-middle SDI | Metabolic risks | 78.70 |
| Deaths | High SDI | Metabolic risks | 77.97 |
| Deaths | Global | Metabolic risks | 77.51 |
| Deaths | Low-middle SDI | Metabolic risks | 77.23 |
| Deaths | Middle SDI | Metabolic risks | 76.96 |
| Deaths | Low SDI | Metabolic risks | 73.50 |
| Deaths | High-middle SDI | High systolic blood pressure | 56.11 |
| Deaths | Low-middle SDI | Behavioral risks | 54.65 |
| Deaths | Low-middle SDI | High systolic blood pressure | 54.11 |
| Deaths | Global | High systolic blood pressure | 53.74 |
| Deaths | Middle SDI | High systolic blood pressure | 53.48 |
| Deaths | Low SDI | Environmental/occupational risks | 53.47 |
| Deaths | Low SDI | Behavioral risks | 53.05 |
| Deaths | Middle SDI | Behavioral risks | 52.41 |
| Deaths | High-middle SDI | Behavioral risks | 52.29 |
| Deaths | Global | Behavioral risks | 51.79 |
| Deaths | High SDI | High systolic blood pressure | 50.66 |
| Deaths | Low SDI | High systolic blood pressure | 50.55 |
| Deaths | Low-middle SDI | Environmental/occupational risks | 47.97 |
| Deaths | Low SDI | Dietary risks | 46.62 |
| Deaths | High SDI | Behavioral risks | 46.09 |
| Deaths | Low SDI | Air pollution | 44.86 |
| Deaths | Low SDI | Particulate matter pollution | 44.86 |
| Deaths | Low-middle SDI | Dietary risks | 44.31 |
| Deaths | Global | Dietary risks | 41.28 |
| Deaths | High-middle SDI | Dietary risks | 41.07 |
| Deaths | Middle SDI | Dietary risks | 40.86 |
| Deaths | Middle SDI | Environmental/occupational risks | 40.74 |
| Deaths | Low-middle SDI | Air pollution | 38.34 |
| Deaths | Low-middle SDI | Particulate matter pollution | 38.34 |
| Deaths | High SDI | Dietary risks | 37.36 |
| Deaths | Global | Environmental/occupational risks | 36.43 |
| Deaths | Low SDI | Household air pollution from solid fuels | 35.11 |
| Deaths | Middle SDI | Air pollution | 31.44 |
| Deaths | Middle SDI | Particulate matter pollution | 31.44 |
| Deaths | High-middle SDI | Environmental/occupational risks | 30.68 |
| Deaths | High-middle SDI | High LDL cholesterol | 29.12 |
| Deaths | High SDI | High LDL cholesterol | 27.84 |
| Deaths | Global | High LDL cholesterol | 27.41 |
| Deaths | Global | Particulate matter pollution | 27.22 |
| Deaths | Global | Air pollution | 27.22 |
| Deaths | Middle SDI | High LDL cholesterol | 27.10 |
| Deaths | Low-middle SDI | High LDL cholesterol | 25.89 |
| Deaths | Middle SDI | Ambient particulate matter pollution | 24.96 |
| Deaths | Low SDI | High LDL cholesterol | 23.87 |
| Deaths | High-middle SDI | Air pollution | 21.71 |
| Deaths | High-middle SDI | Particulate matter pollution | 21.71 |
| Deaths | Low-middle SDI | Household air pollution from solid fuels | 20.39 |
| Deaths | High-middle SDI | Ambient particulate matter pollution | 20.15 |
| Deaths | High SDI | Environmental/occupational risks | 20.08 |
| Deaths | Global | Ambient particulate matter pollution | 19.16 |
| Deaths | High SDI | High fasting plasma glucose | 18.36 |
| Deaths | High SDI | Kidney dysfunction | 18.32 |
| Deaths | Middle SDI | Tobacco | 18.18 |
| Deaths | High-middle SDI | Tobacco | 18.07 |
| Deaths | Low-middle SDI | Ambient particulate matter pollution | 17.95 |
| Deaths | Low-middle SDI | Tobacco | 17.41 |
| Deaths | Global | Tobacco | 17.01 |
| Deaths | Low-middle SDI | High fasting plasma glucose | 16.88 |
| Deaths | Global | Kidney dysfunction | 16.15 |
| Deaths | Low-middle SDI | Kidney dysfunction | 16.15 |
| Deaths | Global | High fasting plasma glucose | 15.82 |
| Deaths | Middle SDI | Kidney dysfunction | 15.77 |
| Deaths | Middle SDI | High fasting plasma glucose | 15.66 |
| Deaths | High-middle SDI | Kidney dysfunction | 15.47 |
| Deaths | Low SDI | Kidney dysfunction | 15.21 |
| Deaths | Low SDI | High fasting plasma glucose | 14.72 |
| Deaths | High SDI | Tobacco | 14.01 |
| Deaths | High-middle SDI | High fasting plasma glucose | 14.01 |
| Deaths | Low-middle SDI | Smoking | 13.95 |
| Deaths | Middle SDI | Smoking | 13.82 |
| Deaths | High-middle SDI | Smoking | 13.73 |
| Deaths | Global | Smoking | 13.31 |
| Deaths | High SDI | High body-mass index | 13.07 |
| Deaths | High-middle SDI | Diet low in whole grains | 13.06 |
| Deaths | Low SDI | Diet low in whole grains | 12.34 |
| Deaths | Low SDI | Tobacco | 12.16 |
| Deaths | High SDI | Smoking | 11.98 |
| Deaths | High-middle SDI | High body-mass index | 11.97 |
| Deaths | Low SDI | Diet low in seafood omega-3 fatty acids | 11.53 |
| Deaths | Global | Diet low in whole grains | 11.49 |
| Deaths | Low-middle SDI | Diet low in whole grains | 10.99 |
| Deaths | High SDI | Air pollution | 10.93 |
| Deaths | High SDI | Particulate matter pollution | 10.93 |
| Deaths | High SDI | Ambient particulate matter pollution | 10.87 |
| Deaths | Low SDI | Lead exposure | 10.73 |
| Deaths | Low SDI | Other environmental risks | 10.73 |
| Deaths | Middle SDI | Diet low in whole grains | 10.71 |
| Deaths | High SDI | Diet low in whole grains | 10.55 |
| Deaths | Global | High body-mass index | 10.23 |
| Deaths | Low-middle SDI | Lead exposure | 9.91 |
| Deaths | Low-middle SDI | Other environmental risks | 9.91 |
| Deaths | Low SDI | Ambient particulate matter pollution | 9.75 |
| Deaths | Low SDI | Smoking | 9.74 |
| Deaths | Low SDI | Diet low in fruits | 9.65 |
| Deaths | Low SDI | Diet low in polyunsaturated fatty acids | 9.17 |
| Deaths | Low-middle SDI | Diet low in fruits | 9.14 |
| Deaths | Middle SDI | High body-mass index | 8.91 |
| Deaths | Middle SDI | Diet high in sodium | 8.86 |
| Deaths | Low-middle SDI | Diet low in polyunsaturated fatty acids | 8.69 |
| Deaths | Low-middle SDI | Diet low in seafood omega-3 fatty acids | 8.48 |
| Deaths | Low-middle SDI | Diet low in nuts and seeds | 8.45 |
| Deaths | High-middle SDI | Diet high in sodium | 8.43 |
| Deaths | Low-middle SDI | High body-mass index | 8.43 |
| Deaths | Global | Household air pollution from solid fuels | 8.05 |
| Deaths | Middle SDI | Diet low in polyunsaturated fatty acids | 7.94 |
| Deaths | Middle SDI | Lead exposure | 7.66 |
| Deaths | Middle SDI | Other environmental risks | 7.66 |
| Deaths | High-middle SDI | Diet low in polyunsaturated fatty acids | 7.65 |
| Deaths | Low SDI | Diet low in nuts and seeds | 7.61 |
| Deaths | Global | Diet low in polyunsaturated fatty acids | 7.50 |
| Deaths | Global | Diet high in sodium | 7.46 |
| Deaths | High-middle SDI | Non-optimal temperature | 7.13 |
| Deaths | High SDI | Non-optimal temperature | 7.07 |
| Deaths | Global | Non-optimal temperature | 6.80 |
| Deaths | High-middle SDI | Diet low in nuts and seeds | 6.80 |
| Deaths | Global | Other environmental risks | 6.78 |
| Deaths | Global | Lead exposure | 6.78 |
| Deaths | Low-middle SDI | Non-optimal temperature | 6.67 |
| Deaths | High-middle SDI | Low temperature | 6.63 |
| Deaths | Middle SDI | Non-optimal temperature | 6.63 |
| Deaths | High SDI | Low temperature | 6.55 |
| Deaths | Global | Diet low in nuts and seeds | 6.54 |
| Deaths | Middle SDI | Household air pollution from solid fuels | 6.47 |
| Deaths | Middle SDI | Diet low in seafood omega-3 fatty acids | 6.30 |
| Deaths | Global | Diet low in seafood omega-3 fatty acids | 6.25 |
| Deaths | High SDI | Diet high in sodium | 6.08 |
| Deaths | Middle SDI | Diet low in nuts and seeds | 6.05 |
| Deaths | Low-middle SDI | Diet low in fiber | 5.89 |
| Deaths | Low SDI | High body-mass index | 5.87 |
| Deaths | Global | Diet low in fruits | 5.70 |
| Deaths | Global | Low temperature | 5.69 |
| Deaths | Low-middle SDI | Diet high in sodium | 5.63 |
| Deaths | Middle SDI | Diet low in fruits | 5.53 |
| Deaths | Low SDI | Non-optimal temperature | 5.51 |
| Deaths | Low SDI | Diet low in vegetables | 5.50 |
| Deaths | Middle SDI | Low temperature | 5.44 |
| Deaths | High-middle SDI | Secondhand smoke | 5.07 |
| Deaths | Middle SDI | Secondhand smoke | 5.04 |
| Deaths | Low SDI | Diet low in fiber | 5.03 |
| Deaths | High-middle SDI | Lead exposure | 4.92 |
| Deaths | High-middle SDI | Other environmental risks | 4.92 |
| Deaths | High SDI | Diet low in fiber | 4.77 |
| Deaths | High SDI | Diet low in seafood omega-3 fatty acids | 4.77 |
| Deaths | High SDI | Diet low in legumes | 4.73 |
| Deaths | High-middle SDI | Diet low in seafood omega-3 fatty acids | 4.65 |
| Deaths | Low SDI | Diet high in sodium | 4.64 |
| Deaths | High SDI | Diet low in polyunsaturated fatty acids | 4.56 |
| Deaths | High SDI | Diet low in nuts and seeds | 4.50 |
| Deaths | Global | Diet low in fiber | 4.42 |
| Deaths | Low-middle SDI | Low temperature | 4.37 |
| Deaths | High-middle SDI | Diet low in legumes | 4.34 |
| Deaths | Global | Secondhand smoke | 4.31 |
| Deaths | Middle SDI | Diet low in fiber | 4.24 |
| Deaths | Low SDI | Low temperature | 4.11 |
| Deaths | High-middle SDI | Diet low in fruits | 4.10 |
| Deaths | Low-middle SDI | Secondhand smoke | 4.03 |
| Deaths | Global | Diet low in legumes | 4.02 |
| Deaths | Low SDI | Diet low in legumes | 3.95 |
| Deaths | High SDI | Diet low in fruits | 3.70 |
| Deaths | Low-middle SDI | Diet low in legumes | 3.61 |
| Deaths | High SDI | Lead exposure | 3.60 |
| Deaths | High SDI | Other environmental risks | 3.60 |
| Deaths | Middle SDI | Diet low in legumes | 3.60 |
| Deaths | Low-middle SDI | Diet low in vegetables | 3.50 |
| Deaths | High-middle SDI | Diet low in fiber | 3.36 |
| Deaths | High SDI | Diet high in red meat | 3.25 |
| Deaths | Middle SDI | Low physical activity | 3.06 |
| Deaths | High SDI | Diet high in processed meat | 2.91 |
| Deaths | High-middle SDI | Low physical activity | 2.85 |
| Deaths | Global | Low physical activity | 2.78 |
| Deaths | Low SDI | Secondhand smoke | 2.76 |
| Deaths | High SDI | Low physical activity | 2.66 |
| Deaths | Low-middle SDI | Low physical activity | 2.64 |
| Deaths | High-middle SDI | Diet high in red meat | 2.59 |
| Deaths | Low-middle SDI | High temperature | 2.44 |
| Deaths | High SDI | Secondhand smoke | 2.39 |
| Deaths | Global | Diet low in vegetables | 2.35 |
| Deaths | High SDI | Diet low in vegetables | 2.22 |
| Deaths | Low-middle SDI | Diet high in trans fatty acids | 2.19 |
| Deaths | Middle SDI | Diet low in vegetables | 2.14 |
| Deaths | Global | Diet high in red meat | 1.85 |
| Deaths | Middle SDI | Diet high in red meat | 1.68 |
| Deaths | Low SDI | Low physical activity | 1.57 |
| Deaths | High-middle SDI | Household air pollution from solid fuels | 1.55 |
| Deaths | Low SDI | High temperature | 1.47 |
| Deaths | High-middle SDI | Diet low in vegetables | 1.34 |
| Deaths | Middle SDI | High temperature | 1.28 |
| Deaths | Global | High temperature | 1.18 |
| Deaths | Low SDI | Diet high in trans fatty acids | 1.09 |
| Deaths | Middle SDI | Diet high in trans fatty acids | 1.00 |
| Deaths | High-middle SDI | Diet high in processed meat | 0.93 |
| Deaths | Global | Diet high in trans fatty acids | 0.89 |
| Deaths | Global | Diet high in processed meat | 0.80 |
| Deaths | High SDI | High temperature | 0.57 |
| Deaths | High-middle SDI | High temperature | 0.54 |
| Deaths | High SDI | Diet high in trans fatty acids | 0.42 |
| Deaths | Low-middle SDI | Diet high in red meat | 0.24 |
| Deaths | High-middle SDI | Diet high in trans fatty acids | 0.16 |
| Deaths | Low SDI | Diet high in processed meat | 0.15 |
| Deaths | High SDI | Diet high in sugar-sweetened beverages | 0.11 |
| Deaths | Low-middle SDI | Diet high in processed meat | 0.11 |
| Deaths | Middle SDI | Diet high in processed meat | 0.09 |
| Deaths | Global | Diet high in sugar-sweetened beverages | 0.06 |
| Deaths | High SDI | Household air pollution from solid fuels | 0.06 |
| Deaths | High-middle SDI | Diet high in sugar-sweetened beverages | 0.05 |
| Deaths | Middle SDI | Diet high in sugar-sweetened beverages | 0.05 |
| Deaths | Low SDI | Diet high in red meat | 0.04 |
| Deaths | Low-middle SDI | Diet high in sugar-sweetened beverages | 0.04 |
| Deaths | Low SDI | Diet high in sugar-sweetened beverages | 0.02 |
| Deaths | Low-middle SDI | Alcohol use | -0.65 |
| Deaths | Low SDI | Alcohol use | -0.98 |
| Deaths | Middle SDI | Alcohol use | -1.06 |
| Deaths | Global | Alcohol use | -1.26 |
| Deaths | High-middle SDI | Alcohol use | -1.33 |
| Deaths | High SDI | Alcohol use | -2.30 |

**Abbreviations:** SDI, socio-demographic index; DALYs, disability-adjusted life years.
